# Incisional negative pressure wound therapy for the prevention of surgical site infection: an up-to-date meta-analysis and trial sequential analysis

**DOI:** 10.1016/j.eclinm.2023.102105

**Published:** 2023-07-24

**Authors:** Hannah Groenen, Hasti Jalalzadeh, Dennis R. Buis, Yasmine E.M. Dreissen, Jon H.M. Goosen, Mitchel Griekspoor, Wouter J. Harmsen, Frank F.A. IJpma, Maarten J. van der Laan, Roald R. Schaad, Patrique Segers, Wil C. van der Zwet, Stijn W. de Jonge, Ricardo G. Orsini, Anne M. Eskes, Niels Wolfhagen, Marja A. Boermeester

**Affiliations:** a Department of Surgery, Amsterdam UMC Location University of Amsterdam, Meibergdreef 9, Amsterdam, Netherlands; b Amsterdam Gastroenterology Endocrinology & Metabolism, Amsterdam, Netherlands; c Dutch National Guideline Group for Prevention of Postoperative Surgical Site Infections, Netherlands; d Department of Neurosurgery, Amsterdam UMC Location University of Amsterdam, Meibergdreef 9, Amsterdam, Netherlands; e Department of Orthopedic Surgery, Sint Maartenskliniek, Ubbergen, Netherlands; f Dutch Association of Medical Specialists, Utrecht, Netherlands; g Division of Trauma Surgery, Department of Surgery, University Medical Center Groningen, Groningen, Netherlands; h Department of Surgery, University Medical Center Groningen, Groningen, Netherlands; i Department of Anesthesiology, Leiden University Medical Centre, Leiden, Netherlands; j Dutch Association of Anesthesiology (NVA), Netherlands; k Department of Cardiothoracic Surgery, Maastricht University Medical Center+, Maastricht, Netherlands; l Department of Medical Microbiology, Infectious Diseases and Infection Prevention, Maastricht University Medical Center, Maastricht, Netherlands; m Department of Surgery, Maastricht University Medical Center+, Maastricht, Netherlands; n Faculty of Health, Center of Expertise Urban Vitality, Amsterdam University of Applied Sciences, Amsterdam, Netherlands; o Menzies Health Institute Queensland and School of Nursing and Midwifery, Griffith University, Gold Coast, Australia

**Keywords:** Surgical site infections, Prevention, Incisional negative pressure wound therapy, iNPWT

## Abstract

**Background:**

The evidence on prophylactic use of negative pressure wound therapy on primary closed incisional wounds (iNPWT) for the prevention of surgical site infections (SSI) is confusing and ambiguous. Implementation in daily practice is impaired by inconsistent recommendations in current international guidelines and published meta-analyses. More recently, multiple new randomised controlled trials (RCTs) have been published. We aimed to provide an overview of all meta-analyses and their characteristics; to conduct a new and up-to-date systematic review and meta-analysis and Grading of Recommendations Assessment, Development and Evaluation (GRADE) assessment; and to explore the additive value of new RCTs with a trial sequential analysis (TSA).

**Methods:**

PubMed, Embase and Cochrane CENTRAL databases were searched from database inception to October 24, 2022. We identified existing meta-analyses covering all surgical specialties and RCTs studying the effect of iNPWT compared with standard dressings in all types of surgery on the incidence of SSI, wound dehiscence, reoperation, seroma, hematoma, mortality, readmission rate, skin blistering, skin necrosis, pain, and adverse effects of the intervention. We calculated relative risks (RR) with corresponding 95% confidence intervals (CI) using a Mantel-Haenszel random-effects model. We assessed publication bias with a comparison-adjusted funnel plot. TSA was used to assess the risk of random error. The certainty of evidence was evaluated using the Cochrane Risk of Bias-2 (RoB2) tool and GRADE approach. This study is registered with PROSPERO, CRD42022312995.

**Findings:**

We identified eight previously published general meta-analyses investigating iNPWT and compared their results to present meta-analysis. For the updated systematic review, 57 RCTs with 13,744 patients were included in the quantitative analysis for SSI, yielding a RR of 0.67 (95% CI: 0.59–0.76, *I*^2^ = 21%) for iNPWT compared with standard dressing. Certainty of evidence was high. Compared with previous meta-analyses, the RR stabilised, and the confidence interval narrowed. In the TSA, the cumulative Z-curve crossed the trial sequential monitoring boundary for benefit, confirming the robustness of the summary effect estimate from the meta-analysis.

**Interpretation:**

In this up-to-date meta-analysis, GRADE assessment shows high-certainty evidence that iNPWT is effective in reducing SSI, and uncertainty is less than in previous meta-analyses. TSA indicated that further trials are unlikely to change the effect estimate for the outcome SSI; therefore, if future research is to be conducted on iNPWT, it is crucial to consider what the findings will contribute to the existing robust evidence.

**Funding:**

Dutch Association for Quality Funds Medical Specialists.

**Research in context**

**Evidence before this study**

Evidence from previous randomised controlled trials (RCTs) and meta-analyses seems contradictive and current international guidelines and published meta-analyses give inconsistent recommendations. Some research suggests that negative pressure wound therapy (iNPWT) on primary closed incisional wounds effectively reduces the risk of surgical site infections (SSI), while others do not. We searched Medline (PubMed); Excerpta Medica Database (EMBASE) and Cochrane Central Register of Controlled Trials (CENTRAL) for systematic reviews, meta-analyses, and current international guidelines for the prevention of SSI with the search terms “surgical site infection”, “post-operative wound complication”, “wound dehiscence”, “hematoma”, “seroma”, “skin necrosis” and “negative pressure wound therapy”. The World Health Organization (WHO) guideline published in 2018 suggests the use of iNPWT in high-risk wounds with an overall low quality of evidence, based on six RCTs and 15 observational studies. The National Institute for Health and Care Excellence (NICE) published a medical technologies guidance in 2019 making a recommendation only on the PICO device in high-risk patients. The Centers for Disease Control and Prevention (CDC) do not mention iNPWT in their current guideline. Several previous meta-analyses show moderate and high certainty evidence that iNPWT reduces the risk of SSI compared with standard dressings. The most recent Cochrane Review on this topic found moderate certainty evidence in favour of iNPWT but missed newly published RCTs. Available meta-analyses have not incorporated these RCTs in quantitative analyses and therefore lag behind.

**Added value of this study**

Despite existing evidence on the effectiveness of iNPWT for the prevention of SSI, iNPWT is still not standard practice. This situation is possibly due to the ambiguity of recommendations from trials and guidelines, and the former paucity of subspecialty evidence on which surgeons usually focus. We provide an overview of all meta-analyses and their characteristics comparing the efficacy of iNPWT to standard dressings on the incidence of SSI and we conduct an up-to-date systematic review and meta-analysis including also the recent RTCs. Grading of Recommendations Assessment, Development and Evaluation (GRADE) assessment shows high-certainty evidence that iNPWT is effective in reducing SSI in patients undergoing a surgical procedure of any wound classification. This result is substantiated in the sensitivity analyses of only studies with low risk of bias, and studies without funding or involvement of the industry. Compared with previous meta-analyses the RR stabilised and the confidence interval narrowed, indicating incremental certainty of the evidence. Newly, we performed a trial sequential analysis (TSA) to explore the additive value of new randomised controlled trials. The cumulative Z-curve crossed the trial sequential monitoring boundary for benefit, indicating that future randomised controlled trials are unlikely to change the effect estimate for the outcome SSI.

**Implications of all the available evidence**

The findings of this up-to-date meta-analysis of 57 RCTs comprising 13,744 patients show, with high-quality evidence, the significant benefit of iNPWT over standard dressings for the prevention of SSI in all wound classifications. In addition, TSA indicated that new studies are unlikely to change the effect estimate.

## Introduction

Surgical site infection (SSI) is a common postoperative complication and causes increased morbidity, mortality and healthcare costs.^1^^,^^2^ Furthermore, other wound complications such as wound dehiscence, hematoma, seroma, and skin necrosis occur frequently. The evidence on prophylactic use of negative pressure wound therapy on primary closed incisional wounds (iNPWT) for the prevention of postoperative wound complications, including SSI, is confusing and ambiguous. It is hypothesised that iNPWT reduces bacterial contamination, exudate and oedema, promotes lymphatic and local blood flow, and stimulates tissue granulation.^3^

iNPWT has been the subject of multiple randomised controlled trials (RCTs), systematic reviews and meta-analyses.^4^, ^5^, ^6^, ^7^, ^8^, ^9^, ^10^ The current international guidelines^11^, ^12^, ^13^ for the prevention of SSI and meta-analyses make inconsistent recommendations. Unfortunately, there is great clinical and methodological heterogeneity between these guidelines and articles, which creates confusion and ambiguity, potentially impairing implementation. The World Health Organization (WHO) guideline [published 2018] suggests the use of iNPWT in high-risk wounds with an overall low quality evidence, based on six RCTs and 15 observational studies.^11^ The National Institute for Health and Care Excellence (NICE) only published a medical technologies guidance in 2019, recommending the PICO device in high-risk patients,^12^ whereas the Centers for Disease Control and Prevention (CDC) do not mention iNPWT in their current guideline.^13^ Several previous meta-analyses show moderate and high certainty evidence that iNPWT reduces the risk of SSI compared with standard dressings.^5^^,^^6^^,^^8^^,^^14^ The most recent Cochrane Review on this topic found moderate certainty evidence in favour of iNPWT.^10^ Despite this evidence, the use of iNPWT is still not standard practice. This is possibly due to the ambiguity of recommendations from trials and guidelines, and the focus of surgeons on their own subspecialty which causes them to disregard results in general populations. However, there is no biological reason to expect a difference in effect between different types of surgery.^15^ Recently, new RCTs have been published. Available meta-analyses have not incorporated these RCTs in quantitative analyses and therefore lag behind.

In this study, we have multiple aims. First, we provide an overview of all available meta-analyses and their characteristics and explore clinical and methodological discrepancies of current meta-analyses on the prophylactic use of iNPWT. Secondly, we conduct a new and up-to-date systematic review, meta-analysis and Grading of Recommendations Assessment, Development and Evaluation (GRADE) assessment. Thirdly, we explore additive value of new future RCTs with a trial sequential analysis (TSA).

## Methods

### Search strategy and selection criteria

Present systematic review and meta-analysis is registered in the PROSPERO database (CRD42022312995) and is reported according to the Preferred Reporting Items for Systematic Reviews and Meta-analysis (PRISMA) statement.^16^

We identified existing systematic reviews and meta-analyses of RCTs, including those conducted for guideline development, comparing iNPWT with standard dressings in all types of surgery. For the systematic review and meta-analysis, unpublished or published RCTs comparing iNPWT with standard dressings on closed incisional wounds in adult patients undergoing any type of surgery were included. If the manuscript of a conference abstract was unavailable at the date of the systematic search, availability of the full manuscript of included conference abstracts published after the database search date was checked previously to publication of this study. If available, the full manuscript was evaluated. RCTs had to report on at least one of the following: SSI, wound dehiscence, reoperation, seroma, hematoma, mortality, readmission, skin blistering or skin necrosis, either as primary, or secondary outcome. Studies investigating NPWT in open wounds, skin grafts, and ulcers were excluded. Furthermore, we excluded animal studies, non-randomised studies, within-subject experimental designs, and studies investing surgeries performed outside the operating theatre. There were no restrictions on the year of publication or language.

We updated the systematic literature search of the previous systematic review performed by our research group.^5^ A clinical librarian was consulted to aid the search. The search was carried out in Medline (PubMed); Excerpta Medica Database (EMBASE) and Cochrane Central Register of Controlled Trials (CENTRAL) from inception to October 24, 2022. Search terms included: “surgical site infection”, “post-operative wound complication”, “wound dehiscence”, “hematoma”, “seroma”, “skin necrosis”, “negative pressure wound therapy”. We identified additional articles by backward and forward citation tracking of previously published systematic reviews, meta-analyses and included RCTs. The complete search can be found in Appendix 1.

Title and abstract screening and full text review of potential eligible studies was conducted by two reviewers (HG and HJ) independently. Discrepancies between the reviewers were settled through discussion and, if necessary, the senior author (MAB) was consulted.

### Data analysis

For the overview of all available evidence, the following data was extracted from the meta-analyses using a standardised form: year of publication, included studies, total number of patients and events in the study arms, effect measure, GRADE assessment and heterogeneity (*I*^2^). To compare results of the meta-analyses, when effect measures were expressed in odds ratio, we calculated relative risks (RR) and corresponding 95% confidence intervals (CI).

For the meta-analysis, a predefined table was used to extract the following data from the RCTs by two review authors (HG and HJ) independently: author, year, country, primary and secondary outcomes, number of patients in each arm, type of surgery, CDC wound classification (clean, clean-contaminated, contaminated and dirty),^17^ type, duration and pressure of iNPWT, type of standard dressing used as control treatment, involvement of the industry, administration of surgical antimicrobial prophylaxis, number and type of SSI, definition of SSI, pain, and adverse events. We contacted corresponding authors in case information was unclear or missing. When the pressure of iNPWT was not mentioned, we assumed that the PICO system (Smith & Nephew, Hull, United Kingdom) always delivers negative pressure at −80 mmHg, and the PREVENA system (KCI, San Antonio, Texas, United States of America) at −125 mmHg.

The primary outcome measure was the incidence of SSI (ie, superficial, deep and organ space), defined at the author's discretion. Secondary outcomes were wound dehiscence, reoperation rate, seroma, hematoma, mortality, readmission rate, skin blistering, skin necrosis, pain, and adverse effects of the intervention.

We calculated RR, corresponding 95% CI and standard errors for the individual trials. Studies with no events in both arms were excluded from the quantitative analysis.^18^ Meta-analyses were performed using a random-effects model (Mantel-Haenszel). A *p-value* < 0.05 was considered statistically significant. We assessed statistical heterogeneity using *I*^2^ statistic and τ^2^.

Assessment of risk of bias of the eligible trials was appraised independently by two authors (HG and HJ), using the Cochrane Risk of Bias-2 (RoB2) tool.^19^ A comparison-adjusted funnel plot was used to judge small-study effects. Asymmetry of the funnel plot can be caused by publication bias or systematic differences between smaller and larger studies. If there is no asymmetry (no small-study effect), publication bias is less likely.^20^ The Grading of Recommendations, Assessment, Development and Evaluations (GRADE) approach was used to assess the certainty of evidence from the eligible studies by evaluating the following domains: risk of bias, imprecision, inconsistency, indirectness, and publication bias.^21^

A priori planned subgroup analyses were carried out on study level based on *type of surgery* (abdominal, vascular, orthopaedic/trauma, plastic, obstetric, breast, general, and cardiac surgery), *industry involvement* (no involvement of the industry, involvement of the industry without involvement in the design, involvement of the industry in design, or no information), the *pressure of the device* (−80 vs. −125 mmHg vs. cyclic vs. no information on pressure), and a sensitivity analysis *excluding RCTs with a high Risk of Bias*. Differences in subgroup analysis were tested with a chi-squared test. In a meta-regression analysis we examined the effect of the *duration of intended treatment* on the effect sizes, shown in a bubble plot.^22^ The size of the bubbles reflects proportional to the weight that the RCTs received in the analysis.

To assess the robustness for the primary outcome of present meta-analysis, we performed a trial sequential analysis (TSA).^23^ This gives the opportunity to calculate the required information size, a summation of sample sizes from the included trials taking variability into account, and estimate trial sequential monitoring boundaries. The required information size and trial sequential monitoring boundaries were based on a type I error of 5%, a power of 80%, a conservative relative risk reduction (RRR) of 15% (minimal clinical important difference), and an SSI risk in the control group of 11.63% (the incidence as found in this meta-analysis).

Statistical analysis was done using R version 4.0.3 [R Core Team (2016) R: A language and environment for statistical computing; R Foundation for Statistical Computing, Vienna, Austria], using the package “meta”. TSA was performed using TSA program version 0.9 beta (Copenhagen Trial Unit, Centre for Clinical Intervention Research, Rigshospitalet, Copenhagen, Denmark).

### Ethics

All data used in this systematic review and meta-analysis is publicly available, ethics committee approval or patient consent for publication was not needed.

### Role of the funding source

The funder of the study had no role in study design, data collection, data analysis, data interpretation, or writing of the report.

## Results

**Fig. 1:**
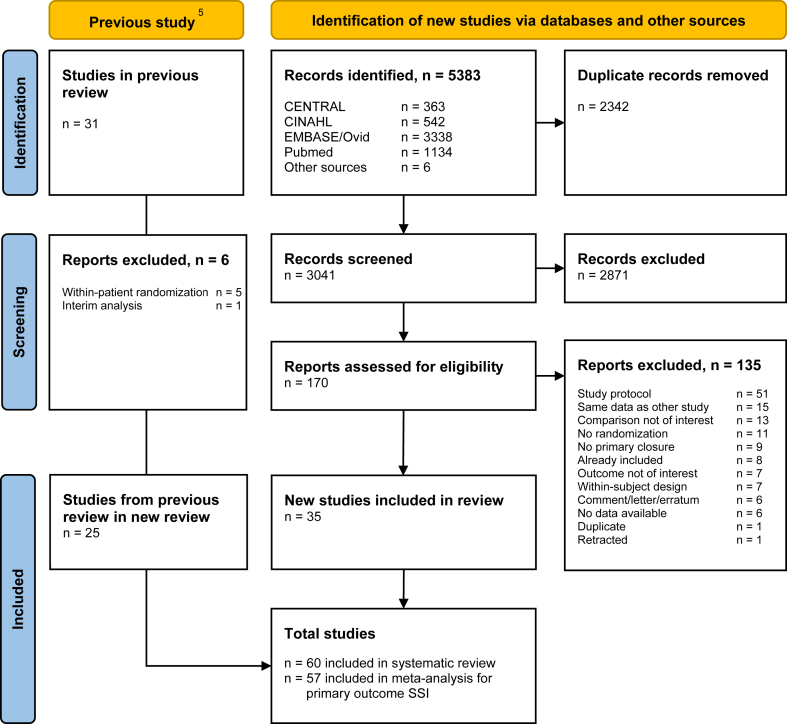
**PRISMA flow chart of study selection**. PRISMA=Preferred Reporting Items for Systematic Reviews and Meta-Analyses; SSI = surgical site infection.

The update of the systematic search provided a total of 5383 records. We screened 3040 studies for title and abstract and reviewed 170 full texts. Six additional records were identified through back- and forward citation tracking. In total, 60 RCTs were included with a total of 13,903 patients for all outcomes. Fig. 1 depicts the systematic review flow chart study selection.

**Fig. 2:**
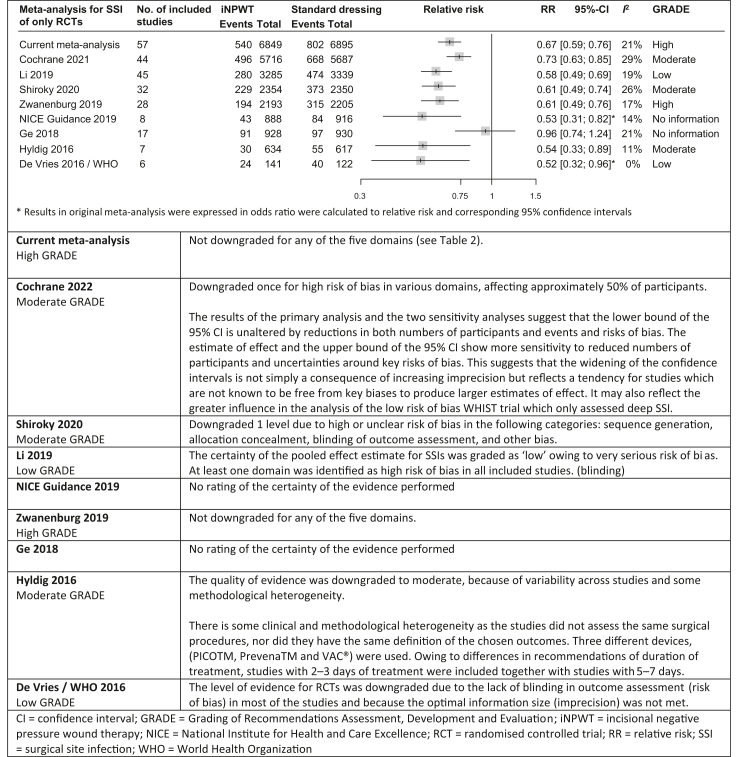
**Comparison of meta-analyses of RCTs and GRADE on the prophylactic use of iNPWT vs. standard dressing**.

We identified eight previously published meta-analyses investigating the effects of iNPWT on SSI in all types of surgery. Two of the included meta-analyses were used for guideline development.^4^^,^^12^ Fig. 2 displays the results of the previous meta-analyses versus the new and up-to-date meta-analysis.

For the primary outcome SSI, an overview of the RCTs included in the updated meta-analysis in comparison with the RCTs included in the previously published meta-analyses is listed in Appendix 2. Six RCTs included in the previous systematic review^5^ were excluded, as one was an interim analysis^24^ of another included study,^25^ and five RCTs had within-subject experimental designs.^26^, ^27^, ^28^, ^29^, ^30^ Reasons for exclusion of full texts can be found in Appendix 3.

The study characteristics of the included RCTs are listed in Appendix 4. We included data of two conference abstracts,^31^^,^^32^ and of one conference abstract the full manuscript recently became available.^33^ iNPWT was compared with standard dressing which varied between studies. Standard dressings used as control varied between studies: gauze-based dressings with or without silver, hydrocolloid-based dressings or silicone gauze were used, or no description was given. Minimum duration of intended iNPWT therapy varied from 2 to 7 days. Definitions for SSI, wound dehiscence, hematoma, seroma, skin necrosis, and the follow-up differed between the included RCTs (Appendix 5).

**Fig. 3:**
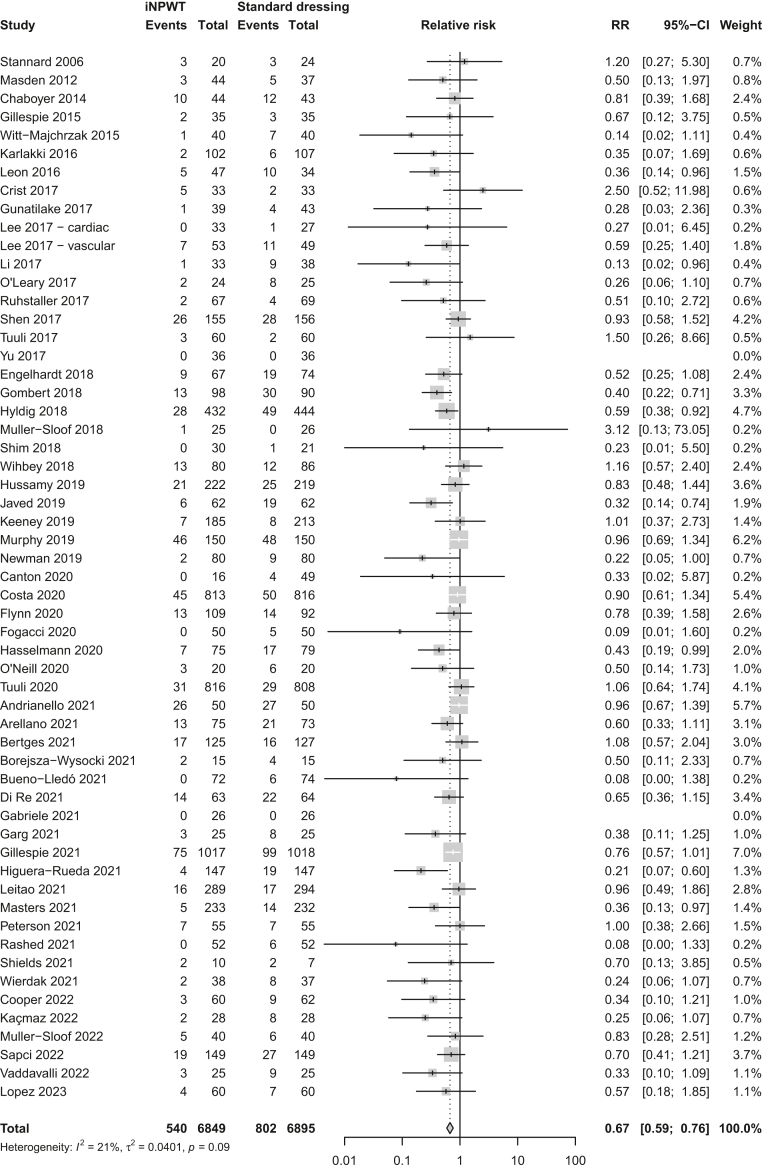
**Forest plot of meta-analysis comparing iNPWT with standard dressing on the risk of SSI**. RR with corresponding 95% CI are shown. CI = confidence interval; iNPWT = incisional negative pressure wound therapy; RR = relative risk; SSI = surgical site infection.

Of the 60 RCTs included in the systematic review, we included 57 RCTs, with a total of 13,744 patients, in the meta-analysis for primary outcome SSI, because three^34^, ^35^, ^36^ RCTs only reported secondary outcomes. The most recent Cochrane Review from 2022 included 44 RCTs with 11,403 patients in the meta-analysis for primary outcome SSI.^10^ Current meta-analysis additionally included 23 RCTs^33^^,^^37^, ^38^, ^39^, ^40^, ^41^, ^42^, ^43^, ^44^, ^45^, ^46^, ^47^, ^48^, ^49^, ^50^, ^51^, ^52^, ^53^, ^54^, ^55^, ^56^, ^57^, ^58^ for SSI that were not included in the Cochrane Review.^10^ We made different methodological choices resulting in the exclusion of ten RCTs^59^, ^60^, ^61^, ^62^, ^63^, ^64^, ^65^, ^66^, ^67^, ^68^ that were included by the Cochrane Review.^10^ Eighteen RCTs^33^^,^^37^, ^38^, ^39^, ^40^, ^41^, ^42^, ^43^, ^44^, ^45^, ^46^^,^^48^^,^^50^^,^^52^, ^53^, ^54^, ^55^^,^^57^ were not yet included in any of the previous published meta-analyses (Appendix 2). Meta-analysis showed a reduction of SSI rate with iNPWT compared with standard dressings (RR 0.67; 95% CI: 0.59–0.76, Fig. 2, Fig. 3, Table 1). Heterogeneity between studies was low (*I*^2^ = 21%, τ^2^ = 0.0401, *p* = 0.09).

**Table 1:** Results of primary outcome, secondary outcomes, sensitivity and subgroup analyses.

	No. of studies	SSIs/participants iNPWT	SSIs/participants standard wound care	RR (95% CI)^a^	GRADE
**Primary outcome**					
**SSI overall**	57	540/6849 (7.9%)	802/6895 (11.6%)	**0.67 (0.59–0.76)**	High
**Type of Surgery**	*p* value for subgroup differences = 0.14				
Abdominal	18	187/1175 (15.9%)	280/1152 (24.3%)	**0.66 (0.54–0.81)**	
Breast	1	0/50 (0%)	5/50 (10.0%)	0.09 (0.01**–**1.60)	
Cardiac	4	1/161 (0.6%)	14/155 (9.0%)	**0.14 (0.03–0.62)**	
General	2	5/54 (9.3%)	7/44 (15.9%)	0.57 (0.19–1.72)	
Obstetric	11	207/3121 (6.6%)	260/3139 (8.3%)	0.82 (0.66–1.03)	
Orthopedic/trauma	12	78/1750 (4.5%)	127/1824 (7.0%)	**0.64 (0.46–0.89)**	
Plastic	3	6/95 (6.3%)	7/87 (8.0%)	0.84 (0.30–2.34)	
Vascular	6	56/443 (12.6%)	102/444 (23.0%)	**0.55 (0.39–0.77)**	
**Industry involvement**	*p* value for subgroup differences = 0.17				
No funding or involvement	16	136/1687 (7.1%)	199/1752 (11.4%)	**0.70 (0.53–0.92)**	
Funding, no involvement	18	300/3612 (8.3%)	400/3606 (11.1%)	**0.74 (0.62–0.88)**	
Funding + involvement	13	74/1129 (6.6%)	132/1156 (11.4%)	**0.59 (0.44–0.80)**	
No information	10	30/421 (7.1%)	71/408 (17.4%)	**0.46 (0.30–0.70)**	
**Risk of Bias**	*p* value for subgroup differences = 0.81				
Low risk of bias	10	105/1373 (7.6%)	149/1373 (10.9%)	**0.72 (0.56–0.91)**	
Some concerns	40	387/4928 (7.9%)	581/4928 (11.8%)	**0.65 (0.55–0.77)**	
Low + Some concerns	50	492/6301 (7.8%)	730/6301 (11.6%)	**0.67 (0.58–0.77)**	
High risk of bias	7	48/548 (8.8%)	72/594 (12.1%)	**0.68 (0.46–0.98)**	
**Pressure of the device**	*p* value for subgroup differences = 0.45				
−125 mmHg	28	280/3131 (8.9%)	406/3128 (13.0%)	**0.69 (0.58–0.82)**	
−80 mmHg	25	247/3616 (6.8%)	372/3677 (10.1%)	**0.67 (0.55–0.81)**	
Cyclic pressure	1	3/20 (15.0%)	3/24 (12.5%)	1.20 (0.27–5.30)	
No information	3	10/82 (12.2%)	21/66 (31.8%)	**0.39 (0.19–0.82)**	
**Secondary outcomes**					
Wound dehiscence	35	332/4417 (7.5%)	387/4450 (8.7%)	0.85 (0.71–1.02)	Moderate
Reoperation	29	91/4629 (2.0%)	106/4691 (2.3%)	0.91 (0.69–1.20)	Low
Seroma	26	108/3444 (3.1%)	134/3440 (3.9%)	0.83 (0.65–1.06)	Moderate
Hematoma	23	32/3419 (0.9%)	47/3408 (1.4%)	0.77 (0.48–1.23)	Low
Mortality	19	32/4052 (0.8%)	34/4053 (0.8%)	0.94 (0.58–1.52)	Low
Readmission	19	113/3241 (3.5%)	114/3246 (3.5%)	0.96 (0.69–1.35)	Low
Skin blistering	15	198/3013 (6.6%)	37/3038 (1.2%)	**5.10 (1.99–13.05)**	Moderate
Necrosis	6	3/444 (0.7%)	14/479 (2.9%)	0.46 (0.14–1.46)	Low

Results are RR with corresponding 95% CI of all included studies on the occurrence of SSI.

CI = confidence interval; GRADE = Grading of Recommendations Assessment, Development and Evaluation; iNPWT = incisional negative pressure wound therapy; RR = relative risk; SSI = surgical site infection.

a Studies with no events in both arms were excluded from quantitative analysis.

The results and forest plots of meta-analyses concerning secondary outcomes are shown in Table 1 and Appendix 6. The secondary outcomes, wound dehiscence, reoperation, seroma, hematoma, mortality, readmission, and skin necrosis, showed no significant results. However, for wound dehiscence and seroma a potential benefit was found [RR 0.85; 95% CI: 0.71–1.02, Appendix 6A and RR 0.83; 95% CI: 0.65–1.06, Appendix 6C], respectively. Skin blistering increased significantly with the use of iNPWT (RR 5.10; 95% CI: 1.99–13.05, Appendix 6G), with high between-study heterogeneity (*I*^2^ = 72%, τ^2^ = 1.6404, *p* < 0.01). Five studies reported no additional treatment was needed for the skin blistering.^25^^,^^45^^,^^69^, ^70^, ^71^, ^72^

Adverse events of the skin related to the study intervention, including skin blistering and pain, were mentioned in 33 RCTs and are listed with detailed explanations when available, in Appendix 7. Five studies reported no adverse events in both study arms.^35^^,^^57^^,^^73^, ^74^, ^75^

The results of the subgroup analyses per type of surgery can be found in Table 1, Appendix 8A and Appendix 8B (subgroup *p =* 0.14). In Appendix 8A, all RCTs assessing iNPWT in orthopaedic and trauma surgery were combined into one group (RR 0.64 95% CI 0.46–0.89) as is commonly done in previous meta-analyses. In Appendix 8B, orthopaedic, and trauma were reported separately; RR 0.41 (95% CI 0.24–0.70), RR 0.84 (95% CI 0.57–1.24). The subgroup analysis based on industry involvement (Table 1, Appendix 8C) showed an RR of 0.70 (95%.CI 0.53–0.92) for studies without any funding or involvement of the industry. Studies with funding but without involvement in study design showed an RR of 0.74 (95% CI 0.62–0.88), while studies with industry funding and involvement in study design showed an RR of 0.59 (95% CI 0.44–0.80). Ten studies reported no information on funding or involvement of the industry (RR 0.46; 95% CI 0.30–0.77). The test for subgroup differences did not indicate a statistically significant subgroup effect for industry involvement (*p* *=* 0.17). Appendix 9 shows an overview of the statements of the included RCTs on industry involvement.

Relative risks for SSI were comparable for negative pressure use of −80 mmHg and −125 mmHg (RR 0.67; 95% CI: 0.55–0.81 and RR 0.69; 95% CI: 0.58–0.82) respectively; subgroup *p* = 0.45), as shown in Table 1 and Appendix 8D. The sensitivity analysis after excluding high risk of bias studies (subgroup *p* = 0.69) and/or studies with some concerns (subgroup *p* = 0.81) showed results comparable to the overall analysis (Table 1, Appendix 8E and 8F). Meta-regression analysis showed that intended duration of treatment is not a significant effect size predictor (*p* = 0.69). Studies with longer intended iNPWT treatment duration were not associated with a difference in SSI, with a regression coefficient of 0.020 (Appendix 10).

**Fig. 4:**
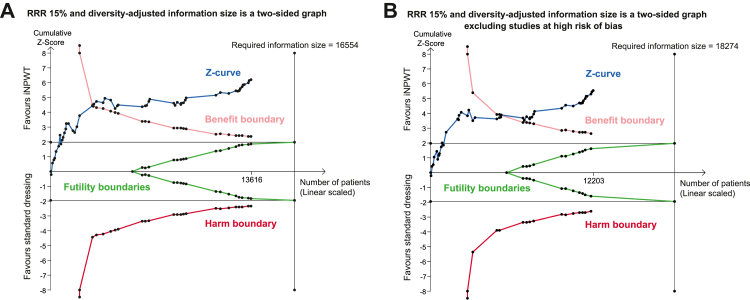
**TSA for primary outcome SSI**. TSA was based on a RRR of 15%, SSI risk in the control group of 11.63%, a type I error of 5% and a type II error of 20%. (A) TSA of all RCTs. (B) TSA excluding studies at high risk of bias. iNPWT = incisional negative pressure wound therapy; RCT = randomised controlled trial; RRR = relative risk reduction; SSI = surgical site infection; TSA = trial sequential analysis.

For TSA for SSI of all trials in the meta-analysis, the required information size was 16,554. The cumulative Z-curve crossed the trial sequential monitoring boundary for benefit, indicating that sufficient evidence exists for a 15% relative risk reduction in SSI by using iNPWT (Fig. 4). This result was substantiated in a sensitivity TSA excluding studies with high risk of bias.

We listed a detailed Risk of Bias assessment in Appendix 11. There were seven RCTs at high risk of bias,^32^^,^^44^^,^^47^^,^^57^^,^^76^, ^77^, ^78^ 43 had some concerns regarding bias^25^^,^^34^, ^35^, ^36^, ^37^, ^38^, ^39^, ^40^^,^^42^^,^^43^^,^^45^^,^^46^^,^^48^^,^^50^^,^^52^^,^^54^^,^^56^^,^^58^^,^^69^, ^70^, ^71^^,^^73^^,^^74^^,^^79^, ^80^, ^81^, ^82^, ^83^, ^84^, ^85^, ^86^, ^87^, ^88^, ^89^, ^90^, ^91^, ^92^, ^93^, ^94^, ^95^, ^96^, ^97^, ^98^ and ten had low risk of bias.^31^^,^^33^^,^^41^^,^^49^^,^^51^^,^^53^^,^^55^^,^^72^^,^^99^^,^^100^

The GRADE assessment for the primary and secondary outcomes is shown in Table 2. For SSI, an overall high certainty of evidence was found as the evidence only came from RCTs and no downgrade was needed on any of the five domains. We found no limitations regarding risk of bias because the results of the sensitivity analysis excluding high risk of bias studies was comparable with the main analysis (Appendix 11). Imprecision was not serious as the 95% confidence interval excluded no effect, and as the trial sequential monitoring boundary for benefit was crossed in the TSA.^101^ There was no serious inconsistency since heterogeneity was low (*I*^2^ = 21%) and confidence intervals overlapped. There was no indirectness. Publication bias was deemed not likely as the comparison-adjusted funnel plot (Appendix 12) showed no asymmetry.

**Table 2:** GRADE assessment for primary and secondary outcomes.

	Certainty assessment							No of patients		Effect		Certainty
	No of studies	Study design	Risk of bias	Inconsistency	Indirectness	Imprecision	Other considerations	iNPWT	Control dressings	Relative (95% CI)	Absolute (95% CI)	
**Primary outcome**												
SSI	57	RCTs	Not serious	Not serious (*I*^2^ = 21%)	Not serious	Not serious	None	540/6849 (7.9%)	802/6895 (11.6%)	RR 0.67 (0.59–0.76)	**38 fewer per 1.000** (from 48 fewer to 28 fewer)	⨁⨁⨁⨁ High
**Secondary outcomes**												
Wound dehiscence	35	RCTs	Not serious	Not serious (*I*^2^ = 13%)	Not serious	**Serious**^a^**(−1)**	None	332/4417 (7.5%)	387/4450 (8.7%)	RR 0.85 (0.71–1.02)	**13 fewer per 1.000** (from 25 fewer to 2 more)	⨁⨁⨁◯ Moderate
Reoperation	29	RCTs	Not serious	Not serious (*I*^2^ = 0%)	Not serious	**Serious**^b^**(−2)**	None	91/4629 (2.0%)	106/4691 (2.3%)	RR 0.91 (0.69–1.20)	**2 fewer per 1.000** (from 7 fewer to 5 more)	⨁⨁◯◯ Low
Seroma	26	RCTs	Not serious	Not serious (*I*^2^ = 0%)	Not serious	**Serious**^a^**(−1)**	None	108/3444 (3.1%)	134/3440 (3.9%)	RR 0.83 (0.65–1.06)	**7 fewer per 1.000** (from 14 fewer to 2 more)	⨁⨁⨁◯ Moderate
Hematoma	23	RCTs	Not serious	Not serious (*I*^2^ = 0%)	Not serious	**Serious**^b^**(−2)**	None	32/3419 (0.9%)	47/3408 (1.4%)	RR 0.77 (0.48–1.23)	**3 fewer per 1.000** (from 7 fewer to 3 more)	⨁⨁◯◯ Low
Mortality	19	RCTs	Not serious	Not serious (*I*^2^ = 0%)	Not serious	**Serious**^b^**(−2)**	None	32/4052 (0.8%)	34/4053 (0.8%)	RR 0.94 (0.58–1.52)	**1 fewer per 1.000** (from 4 fewer to 4 more)	⨁⨁◯◯ Low
Readmission	19	RCTs	Not serious	Not serious (*I*^2^ = 28%)	Not serious	**Serious**^b^**(−2)**	None	113/3241 (3.5%)	114/3246 (3.5%)	RR 0.96 (0.69–1.23)	**1 fewer per 1.000** (from 11 fewer to 8 more)	⨁⨁◯◯ Low
Skin blistering	11	RCTs	Not serious	**Serious (−1)** (*I*^2^ = 72%)	Not serious	Not serious	None	198/3013 (6.6%)	37/3808 (1.2%)	RR 5.10 (1.99–13.05)	**50 more per 1.000** (from 2 more to 156 more)	⨁⨁⨁◯ Moderate
Necrosis	6	RCTs	Not serious	Not serious (*I*^2^ = 0%)	Not serious	**Serious**^b^**(−2)**	None	3/444 (0.7%)	14/479 (2.9%)	RR 0.46 (0.14–1.46)	**16 fewer per 1.000** (from 25 fewer to 13 more)	⨁⨁◯◯ Low

CI = confidence interval; GRADE = Grading of Recommendations Assessment, Development and Evaluation; iNPWT = incisional negative pressure wound therapy; RR = relative risk; SSI = surgical site infection; RCT = randomised controlled trial.

∗ Risk of bias (Appendix 10).

a 95% CI overlaps no effect.

b 95% CI overlaps no effect but fails to exclude considerable benefit or harm (default relative risk reduction >0.20).

## Discussion

This systematic review presents an overview and comparison of all available evidence [both previous meta-analyses and a new up-to-date meta-analysis of all RCTs] regarding the effect of incisional negative pressure wound therapy on the incidence of surgical site infections. This is necessary because current guidelines on the prevention of SSI do not provide updated or unbiased recommendations on iNPWT for the prevention of SSI. We included 23 additional RCTs to this up-to-date meta-analysis compared with the most recent Cochrane Review^10^ and made methodological choices that considered the current high standard of clinical care. Present meta-analysis shows high GRADE evidence that iNPWT is effective in reducing SSI.

Over time, with increasing numbers of studies included in consecutive meta-analyses, the RR has stabilised, and the confidence interval has narrowed, indicating incremental certainty of the evidence (Fig. 2).

A strength of current systematic review is the focus on RCTs across all surgical specialties. This is important because there is no biological rationale why a difference in effect across types of surgery is expected; there are only differences in specific SSI risk. Arbitrary splintering of the available data across surgical subspecialties without evidence for effect modification undermines statistical power and risks spurious results. There is a substantial number of systematic reviews on the use of iNPWT for specific types of surgery, for example obstetric^102^^,^^103^ or vascular surgery.^104^ Considering the high interest in the outcomes per type of surgery, we performed a subgroup analysis. Because of our wide search, yielding over 5000 articles, it is expected that all relevant trials, independent of type of surgery are covered by our search strategy. In subgroup analysis for type of surgery, the effect of iNPWT on the incidence of SSI seemed to be attributable over a broad range of surgical procedures in abdominal, cardiac, orthopaedic/trauma and vascular surgery. When analysing orthopaedic and trauma surgery studies separately, we found a significant benefit for orthopaedic surgery, while the effect in trauma surgery was non-significant with a wide confidence interval, which might be due to a relatively small number of patients in the included studies and/or the more acute nature of the trauma procedures. The results of iNPWT in obstetric surgery also suggested a beneficial effect but remain non-significant with the upper bound of the confidence interval just crossing the 1. For breast, general and plastic surgery, non-significant effects with a wide confidence interval were found, which might be due to imprecision because of the limited number of randomised patients. A priori planned subgroup analysis did not show a clear difference between different levels of negative pressure or different intended duration of iNPWT. We hypothesised that involvement of the industry would reveal a more significant benefit of iNPWT on the incidence of SSI. However, we found a comparable benefit for studies with and studies without involvement of the industry.

Compared with the Cochrane Review^10^ we report different results. The Cochrane Review downgraded their level of evidence to moderate due to risk of bias. They graded down since in their sensitivity analyses of only low risk of bias studies, and combined low and some concerns studies the upper bounds of 95% CI were sensitive to risk of bias. In present review, low risk of bias studies as well as low and some concerns studies combined were not sensitive to risk of bias. In addition, for this review we used the RoB2-tool,^19^ whereas the Cochrane used the first RoB-tool,^105^ which might attribute to different judgements of risk of bias.

Moreover, we employed TSA to assess the risk of random error. This TSA indicates that enough RCTs on this subject have been performed for a RR reduction of 15% in SSI when iNPWT is used, since the cumulative Z-curve crossed the trial sequential monitoring boundary for benefit. The TSA clearly shows that future studies are very unlikely to influence the effect estimate.

For our secondary outcomes, the effect of iNPWT on the incidence of wound dehiscence, seroma, hematoma, mortality, readmission and skin necrosis showed indifferent results. However, for wound dehiscence and seroma a potential benefit was found. For wound dehiscence the RR (0.85; 95% CI 0.71–1.02) was lower compared with the Cochrane Review (RR 0.97; 95% CI 0.82–1.16),^10^ which may indicate that evidence on this outcome may not be settled down when analysis lacks power as fewer studies report this outcome.

We also aimed to explore the potential adverse effects of the skin and pain related to the study intervention. In total, 32 included RCTs reported adverse skin reactions or pain. However, the data were very heterogeneous, not all RCTs mentioned which adverse effects they collected, and no unambiguous definitions of adverse effects were used. Only 15 studies specifically reported skin blistering as an adverse event, with a fivefold increase of skin blistering found in patients with iNPWT compared with standard dressings. Heterogeneity was high (*I*^2^ = 72%), and incidence of skin blistering varied greatly, between 3%^93^ and 28%.^87^ Skin blistering was reported as a minor adverse event that often needed no additional treatment, but should be taken into account and discussed with patients prior to starting iNPWT.

We need to address some limitations of our systematic review and up-to-date meta-analysis. First, there was some methodological and clinical heterogeneity in the included studies, including variation in the definition for the primary outcome SSI. For instance, in studies assessing orthopaedic and trauma patients, the definition for SSI was insufficient or not reported. Instead of the CDC criteria, the fracture-related infection consensus definition and EBJIS periprosthetic joint infection definitions are preferred in these patient groups.^106^, ^107^, ^108^ An important aim of the present study was to take in account all available evidence, therefore we accepted the definition for SSI by authors’ discretion. Secondly, not all studies mentioned the preventive measures for SSI they use as standard perioperative care besides iNPWT. Because all studies were published after the year 2011, we assumed they adhere to best practice guidelines, including adequate timing and (re)dosing perioperative antibiotic prophylaxis and adequate use of skin antiseptics. Thirdly, blinding of the intervention with a (large) device is difficult, if not impossible. However, blinding of outcome assessment is possible, and is considered in one of the domains of the RoB2-tool. When also outcome assessment was not blinded, the study was judged with at least some concerns regarding bias. Thus, in all studies judged with low risk of bias, outcome assessors were blinded for the intervention. In sensitivity analysis of studies with only low risk of bias, we found comparable results to the overall analysis, resulting in high grade evidence.

The present work gives a transparent overview and comparison of all available evidence [both published meta-analyses and RCTs] regarding the effect of incisional negative pressure wound therapy on the incidence of surgical site infections. This new up-to-date meta-analysis provides compelling evidence for the prophylactic use of iNPWT compared with standard dressings for the prevention of SSI in adult patients undergoing any surgical procedure. Moreover, in contrast to previous meta-analyses and guidelines performed by other research groups, the overall level of evidence was graded as high. In addition, TSA shows that additional trials will unlikely shift the existing evidence.

Therefore, when conducting future research on iNPWT, it is crucial to carefully consider what the findings will contribute to the presented robust evidence. In addition, with the high number of ongoing trials we identified, we advise researchers to use clear and standardised definitions and classifications for SSI and other outcomes to minimise heterogeneity and be able to approach the true effect as closely as possible.

## Contributors

HG and HJ contributed equally to this study. HG, HJ, NW and MAB were responsible for the conceptualisation and were actively involved in planning the methodology. HG and HJ contributed equally to the formal analysis, investigation, project administration, visualisation and writing of the original draft. SWJ, RGO, AME, NW and MAB provided critical advice. All authors reviewed, edited, and approved the final version of the manuscript. HG and GJ accessed and verified the underlying data reported in the manuscript. All authors had full access to all the data and responsibility for the decision to submit for publication.

## Data sharing statement

All data is published in this manuscript, the cited manuscripts, or the supplementary appendix. Data can be provided upon request to corresponding authors, and in agreement of terms. No individual participant data was used; we used raw data presented in the cited manuscripts.

## Declaration of interests

MAB reported receiving institutional grants from J&J/Ethicon, 3M, and New Compliance; and being a speaker and/or instructor for J&J/Ethicon, 3M, BD Bard, Gore, Smith & Nephew, TelaBio, Angiodynamics, GDM, Medtronic. JHMG has given presentations for Smith & Nephew, unrelated to NPWT or this publication. RRS was former president of the committee for quality and safety in practice for the Dutch Anesthesiology Society. AME received a European Wound Managament grant. No other disclosures were reported. All other authors report no conflicts of interest.

## Appendix A Supplementary data

Supplements_revision_2_clean_V5
