# Supplementary material for: Incisional negative pressure wound therapy for the prevention of surgical site infection: an up-to-date meta-analysis and trial sequential analysis
Source: eClinicalMedicine. 2023 Jul 24;62:102105. doi: 10.1016/j.eclinm.2023.102105 (PMC10393772; doi:10.1016/j.eclinm.2023.102105)

**Incisional negative pressure wound therapy for the prevention of surgical site infection: an up-to-date meta-analysis and trial sequential analysis**

Hannah Groenen MD, Hasti Jalalzadeh MD, LLM, Dennis R Buis MD, PhD, Yasmine EM Dreissen MD, PhD, Jon HM Goosen MD, PhD, Mitchel Griekspoor MSc, Wouter J Harmsen PhD, Frank FA IJpma MD, PhD, Maarten J van der Laan MD, PhD, Roald R Schaad MD, Patrique Segers MD, PhD, Wil C van der Zwet MD, PhD, Stijn W de Jonge MD, PhD, Ricardo G Orsini MD, PhD, Anne M Eskes PhD, Niels Wolfhagen MD, Prof Marja A Boermeester MD, PhD

*H. Groenen and H. Jalalzadeh contributed equally to this work.

**Supplementary appendix**

# Appendix 1. Search strategy 2

# Appendix 2. Comparison of included studies in and meta-analyses for primary outcome SSI 3

# Appendix 3. Reasons for exclusion after full text review 5

# Appendix 4. Study characteristics 12

# Appendix 5. Definitions of SSI 15

# Appendix 6. Forest plots of secondary outcomes 18

1. Wound dehiscence 18
2. Reoperation 19
3. Seroma 20
4. Hematoma 21
5. Mortality 21
6. Readmission 22
7. Skin blistering 22
8. Necrosis 23

# Appendix 7. Adverse events 23

# Appendix 8. Forest plots of sensitivity and subgroup analyses primary outcome SSI 25

1. Type of surgery – clustering orthopedic and trauma surgery 25
2. Type of surgery – splitting orthopedic and trauma surgery 26
3. Industry involvement 27
4. Pressure of device 28
5. Risk of bias: low/some concerns vs. high risk 29
6. Risk of bias: low vs. some concerns vs. high risk 30

# Appendix 9. Statements on industry involvement 31

# Appendix 10. Bubble plot of meta-regression of intended duration of treatment 33

# Appendix 11. Elaborate Risk of bias assessment 34

# Appendix 12. Comparison-adjusted funnel plot 36

# **Appendix 1. Search strategy**

**PubMed:**

("Negative-Pressure Wound Therapy"[Mesh] OR negative pressure*[tiab] OR NPWT[tiab] OR NPT[tiab] OR vacuum assisted closure*[tiab] OR VAC[tiab] OR V.A.C.[tiab] OR TNP[tiab] OR (topical[tiab] AND subatmospheric pressure*[tiab]) OR TASAP[tiab] OR microdeformational wound therap*[tiab] OR vacuum therap*[tiab] OR closed incision management[tiab] OR surgical incision management[tiab] OR incisional management system*[tiab] OR prevena[tiab])

AND

("Surgical Wound Infection"[Mesh] OR "Surgical Wound Dehiscence"[Mesh] OR "Seroma"[Mesh] OR

"Hematoma"[Mesh] OR "Necrosis"[Mesh] OR surgical site infection*[tiab] OR surgical wound infection*[tiab] OR surgical wound*[tiab] OR surgical incision*[tiab] OR surgical site complication*[tiab] OR surgical site occurrence*[tiab] OR surgical site event*[tiab] OR incision*[tiab] OR prophylactic*[tiab] OR seroma*[tiab] OR necrosis[tiab] OR cellulitis[tiab] OR dehiscence*[tiab] OR hematoma*[tiab])

**Embase (Ovid):**

1. vacuum assisted closure/ or (negative pressure or NPWT or NPT or vacuum assisted closure* or

VAC or TNP or TASAP or microdeformational wound therap* or vacuum therap* or closed incision

management or surgical incision management or incisional management system* or prevena).ti,ab,kw.

or (topical adj6 subatmospheric pressure*).ti,ab,kw.

1. surgical infection/ or wound dehiscence/ or seroma/ or exp hematoma/ or exp necrosis/ or

(surgical site infection* or surgical wound infection* or surgical wound* or surgical incision* or surgical

site complication* or surgical site occurrence* or surgical site event* or incision* or prophylactic* or

seroma* or necrosis or cellulitis or dehiscence* or hematoma*).ti,ab,kw.

1. 1 and 2

**CENTRAL:**

ID Search

#1 negative pressure* or NPWT or NPT or vacuum assisted closure* or VAC or TNP or TASAP or

microdeformational wound therap* or vacuum therap* or closed incision management or surgical

incision management or incisional management system* or prevena:ti,ab,kw (Word variations have

been searched)

#2 surgical site infection* or surgical wound infection* or surgical wound* or surgical incision* or

surgical site complication* or surgical site occurrence* or surgical site event* or incision* or

prophylactic* or seroma* or necrosis or cellulitis or dehiscence* or hematoma*:ti,ab,kw (Word

variations have been searched)

#3 #1 and #2

**CINAHL (Ebsco):**

(MH "Negative Pressure Wound Therapy") OR ( TI ( negative pressure or NPWT or NPT or vacuum

assisted closure* or VAC or TNP or TASAP or microdeformational wound therap* or vacuum therap* or

closed incision management or surgical incision management or incisional management system* or

prevena ) OR AB ( negative pressure or NPWT or NPT or vacuum assisted closure* or VAC or TNP or

TASAP or microdeformational wound therap* or vacuum therap* or closed incision management or

surgical incision management or incisional management system* or prevena ) ) OR ( TI topical N6

subatmospheric pressure* OR AB topical N6 subatmospheric pressure* )

AND

( (MH "Surgical Wound Infection") OR (MH "Surgical Wound Dehiscence") ) OR (MH "Hematoma+") OR

(MH "Necrosis+") OR ( TI ( surgical site infection* or surgical wound infection* or surgical wound* or

surgical incision* or surgical site complication* or surgical site occurrence* or surgical site event* or

incision* or prophylactic* or seroma* or necrosis or cellulitis or dehiscence* or hematoma* ) OR AB (

surgical site infection* or surgical wound infection* or surgical wound* or surgical incision* or surgical

site complication* or surgical site occurrence* or surgical site event* or incision* or prophylactic* or

seroma* or necrosis or cellulitis or dehiscence* or hematoma* ) ).

The search was conducted from inception to 24-10-2022

# **Appendix 2. Comparison of included studies in meta-analyses for primary outcome SSI**

| Cochrane 2021  44 RCTs (n=11,403 patients) in MA | Shiroky 2020  32 RCTs (n=4704 patients) in MA | Li 2019  45 RCTs (n=6624 patients) in MA | Ge 2018  17 RCTs (n=1858 patients) in MA | Hyldig 2016  7 RCTs (n=1251 incisions) in MA | Present MA  56 RCTs (n=13,663 patients) in MA | Reason not included in present MA |
| --- | --- | --- | --- | --- | --- | --- |
|  |  |  |  |  | Lopez 2023 |  |
|  |  |  |  |  | Cooper 2022 |  |
|  |  |  |  |  | Kaçmaz 2022 |  |
|  |  |  |  |  | Muller-Sloof 2022 |  |
|  |  |  |  |  | Sapci 2022 |  |
|  |  |  |  |  | Vaddavalli 2022 |  |
| Andrianello 2021 |  |  |  |  | Andrianello 2021 |  |
|  |  |  |  |  | Arellano 2021 |  |
| Bertges 2021 |  |  |  |  | Bertges 2021 |  |
|  |  |  |  |  | Borejsza-Wysocki 2021 |  |
| Bueno-Lledó 2021 |  |  |  |  | Bueno-Lledó 2021 |  |
|  |  |  |  |  | Di Re 2021 |  |
|  |  |  |  |  | Gabriele 2021^*^ |  |
|  |  |  |  |  | Garg 2021 |  |
| Gillespie 2021 |  |  |  |  | Gillespie 2021 |  |
|  |  |  |  |  | Higuera-Rueda 2021 |  |
| Leitao 2020 |  |  |  |  | Leitao 2021 |  |
|  |  |  |  |  | Masters 2021 |  |
|  |  |  |  |  | Peterson 2021 |  |
|  |  |  |  |  | Rashed 2021 |  |
|  |  |  |  |  | Shields 2021 |  |
| Wierdak 2021 |  |  |  |  | Wierdak 2021 |  |
|  |  |  |  |  | Canton 2020 |  |
| WHIST 2019 |  |  |  |  | Costa 2020 |  |
| Flynn 2020 |  |  |  |  | Flynn 2020 |  |
|  |  | Hasselman 2015 |  |  | Hasselmann 2020 |  |
|  |  |  |  |  | O’Neill 2020 |  |
| Tanaydin 2018 |  |  |  |  |  | Within-subject experimental design |
| Tuuli 2020 | Tuuli 2017 | Tuuli 2017 |  |  | Tuuli 2020 |  |
| Fogacci 2019 |  |  |  |  | Fogacci 2019 |  |
| Hussamy 2017 | Hussamy 2018 | Hussamy 2018 |  |  | Hussamy 2019 |  |
| Javed 2018 | Javed 2019 | Javed 2018 |  |  | Javed 2019 |  |
| Keeney 2019 |  | Keeney 2018 |  |  | Keeney 2019 |  |
|  |  | Ker 2019 |  |  |  | Cost-utility analysis of NPWT in split-skin graft |
| Kuncewitch 2017 |  |  |  |  |  | Subanalysis Shen 2017 |
| Martin 2019 |  |  |  |  |  | Same data as O’Neill 2020 |
| Murphy 2019 | Murphy 2019 | Murphy 2018 |  |  | Murphy 2019 |  |
| Newman 2019 |  | Newman 2018 |  |  | Newman 2019 |  |
|  | Steele 2019^*^ |  |  |  |  | Data only available on ClinicalTrial.gov |
| Bobkiewicz 2018 | Bobkiewicz 2018 | Bobkiewicz 2018 |  |  |  | Same data as Borejsza-Wysocki 2021 |
|  |  | Costa 2018 |  |  |  | Open wound |
| Engelhardt 2018 | Engelhardt 2018 | Engelhardt 2018 |  |  | Engelhardt 2018 |  |
|  | Galiano 2018 | Galiano 2018 |  | Galiano (before publication) |  | Within-subject experimental design |
| Gombert 2018 | Gombert 2018 | Gombert 2018 |  |  | Gombert 2018 |  |
| Hyldig 2019 | Hyldig 2018 | Hyldig 2018 |  |  | Hyldig 2018 |  |
|  | Kwon 2018 | Kwon 2018 |  |  |  | Within-subject experimental design |
|  | Muller-Sloof 2018 | Muller-Sloof 2018 |  |  | Muller-Sloof 2018 |  |
| Shim 2018 |  | Shim 2018 |  |  | Shim 2018 |  |
| Wihbey 2018 | Wihbey 2018 | Wihbey 2018 |  |  | Wihbey 2018 |  |
|  | Pleger 2018 | Pleger 2017 |  |  |  | Within-subject experimental design |
| Crist 2017 | Crist 2017 | Crist 2017 |  | Crist (before publication) | Crist 2017 |  |
| DiMuzio 2017 |  |  |  |  |  | Same data as Kwon 2018 |
| Gunatilake 2017 | Gunatilake 2017 | Gunatilake 2017 |  |  | Gunatilake 2017 |  |
| Lee 2017, card | Lee 2017, card | Lee 2017, card |  |  | Lee 2017, cardiac |  |
| Lee 2017, vasc | Lee 2017, vasc | Lee 2017, vasc |  |  | Lee 2017, vascular |  |
|  | Li 2017 | Li 2017 | Li 2017 |  | Li 2017 |  |
| Lozano-Balderas 2017 |  | Lozano-Balderas 2017 |  |  |  | No primary closure in NPWT group |
|  |  | Mendame-Ehya 2017 |  |  |  | Open wounds |
| O’Leary 2017 | O’Leary 2017 | O’Leary 2017 |  |  | O'Leary 2017 |  |
| Ruhstaller 2017 | Ruhstaller 2017 | Ruhstaller 2017 |  |  | Ruhstaller 2017 |  |
| Shen 2017 | Shen 2017 | Shen 2017 | Shen 2017 |  | Shen 2017 |  |
|  |  | Sibin 2017 |  |  |  | Open wound |
| Tuuli 2017 |  |  |  |  | Tuuli 2017 |  |
|  |  |  | Visser 2017 |  |  | Paediatric population |
|  | Yu 2017^*^ |  | Yu 2017^*^ |  | Yu 2017^*^ |  |
|  |  | Arti 2016 |  |  |  | Open wound |
|  |  |  | Correa 2016 |  |  | Open wound |
| Karlakki 2016 |  | Karlakki 2016 |  | Karlakki (before publication) | Karlakki 2016 |  |
| Leon 2016 | Leon 2016 | Leon 2016 |  |  | Leon 2016 |  |
|  | Sabat 2016 | Sabat 2016 |  |  |  | Analysis by wound |
| Uchino 2016 |  | Uchino 2016 | Uchino 2016 |  |  | No primary closure |
|  |  | Virani 2016 |  |  |  | Open wound |
| NCT01759381 | Bagley 2015 |  |  |  |  | Data only available on ClinicalTrials.gov |
| Gillespie 2015 | Gillespie 2015 | Gillespie 2015 | Gillespie 2015 |  | Gillespie 2015 |  |
| Witt-Majchrzac 2015 | Witt-Majchrzak 2015 | Witt-Majchrzak 2015 |  |  | Witt-Majchrzak 2015 |  |
|  |  | Biter 2014 | Biter 2014 |  |  | Open wound |
| Chaboyer 2014 | Chaboyer 2014 | Chaboyer 2014 | Chaboyer 2014 |  | Chaboyer 2014 |  |
| Crist 2014 |  |  |  |  |  | Conference abstract, same data as Crist 2017 |
|  |  | Gupta 2014 |  |  |  | No primary closure |
|  |  |  | Marston 2014 |  |  | Open wounds |
|  |  |  |  | Grauhan 2013 |  | No RCT |
|  |  | Jayakumar 2013 |  |  |  | Full text not available through library |
|  |  |  | Kakagia 2012 |  |  | No primary closure |
| Masden 2012 |  | Masden 2012 | Masden 2012 | Masden 2012 | Masden 2012 |  |
|  | Stannard 2012 | Stannard 2012 | Stannard 2012 (3x) | Stannard 2012 |  | Analysis by wound |
|  | Howell 2011 | Howell 2011 | Howell 2011 | Howell 2011 |  | Analysis by wound |
|  |  |  | Blume 2007 |  |  | Open wound |
|  | Stannard 2006 |  |  |  | Stannard 2006 |  |
|  |  |  | Vuerstaek 2006 |  |  | Open wound |
|  |  |  | Armstrong 2005 |  |  | Open wound |
| NCT02309944 |  |  |  |  |  | Data only available on ClinicalTrials.gov |
| * Gabriele 2021, Steele 2019 and Yu 2017 reported no events in both arms, and were therefore not included in quantitative analysis | | | | | | |

# **Appendix 3. Reasons for exclusion after full text review**

|  | **Study** | **Reason for exclusion** |
| --- | --- | --- |
| 1 | Abesamis 2019^1^ | No randomization |
| 2 | Achten 2018^2^ | Protocol |
| 3 | ACTRN12619000785101, 2019^3^ | Protocol |
| 4 | Anderson^4^ | Interim analysis of included study (Chaboyer 2014^5^) |
| 5 | Biao 2019^6^ | Comparison not of interest |
| 6 | Brennfleck 2020^7^ | Protocol |
| 7 | Brown 2020^8^ | Protocol |
| 8 | Campolier 2019^9^ | Conference abstract of included study (Costa 2020^10^) |
| 9 | Carrano 2021^11^ | No primary closure |
| 10 | Chaboyer 2021^12^ | Secondary analysis of included study (Gillespie 2021^13^) |
| 11 | Chang 2018^14^ | Comment |
| 12 | Chen 2019^15^ | Comparison not of interest |
| 13 | Chetter 2021^16^ | Protocol |
| 14 | ChiCTR1900022165, 2019^17^ | Protocol |
| 15 | ChiCTR2000034266, 2020 ^18^ | Protocol |
| 16 | Chu 2018^19^ | Comparison not of interest |
| 17 | Clark 2019^20^ | Outcome not of interest |
| 18 | Cocjin 2019^21^ | Comparison not of interest |
| 19 | Cook 2019^22^ | No primary closure |
| 20 | Costa 2018^23^ *(Health Technol Assess.)* | No primary closure |
| 21 | Costa 2018^24^ (*JAMA)* | No primary closure |
| 22 | Costa 2020^25^ *(Health Technol Assess.)* | Same data as included study (Costa 2020^10^*,JAMA)* |
| 23 | CTRI/2019/05/019225, 2019^26^ | Protocol |
| 24 | CTRI/2019/08/020895, 2019^27^ | Protocol |
| 25 | CTRI/2019/09/021388, 2019^28^ | Protocol |
| 26 | Dadras 2022^29^ | Comparison not of interest |
| 27 | Darwisch 2020^30^ | Conference abstract: no data available |
| 28 | Davis 2020^31^ | Comparison not of interest |
| 29 | Di Re 2020^32^ | Protocol |
| 30 | Dondossola 2020^33^ | Letter to the editor |
| 31 | Donlon 2019^34^ | Protocol |
| 32 | DRKS00015136, 2019^35^ | Protocol |
| 33 | DRKS00021494, 2020^36^ | Protocol |
| 34 | Engelhardt 2018^37^ | Already included |
| 35 | Fang 2020^38^ | No randomization |
| 36 | Fernandes 2021^39^ | Conference abstract: no randomisation |
| 37 | Ferrando 2021^40^ | Conference abstract: trial protocol |
| 38 | Fogacci 2019^41^ | Conference abstract of included study (Fogacci 2019^42^) |
| 39 | Galiano 2018^43^ | Within-subject experimental design |
| 40 | Gombert 2018^44^ | Already included |
| 41 | Gombert 2019^45^ | No randomization |
| 42 | Gombert 2020^46^ | Erratum |
| 43 | Gonzalez 2020^47^ | Conference abstract: no data available |
| 44 | Haddad 2021^48^ | Conference abstract of ongoing trial (NCT03773575) |
| 45 | Halama 2019^49^ | Outcome not of interest |
| 46 | Hasselmann 2019^50^ | Conference abstract of Hasselmann 2020^52^ (*Ann Surg.)* |
| 47 | Hasselmann 2020^51^ (*Surg Infect. [Larchmt])* | Same data as Hasselmann 2020^52^ (*Ann Surg.)* |
| 48 | Howell 2011^53^ | Within-subject experimental design |
| 49 | Hyldig 2019^54^ | Already included |
| 50 | Hyldig 2019^55^ | Outcome not of interest |
| 51 | Jaimes 2020^56^ | Conference abstract: outcome not of interest |
| 52 | Javed 2019^57^ | Already included |
| 53 | Jenkins 2022^58^ | Outcome not of interest |
| 54 | Jørgensen 2018*^59^* | Protocol |
| 55 | BJOG. 2019 Apr;126(5):636 (*No author listed) ^60^* | Comment |
| 56 | KCT0004063, 2019^61^ | Protocol |
| 57 | Kim 2020^62^ | Protocol |
| 58 | Knight 2019^63^ | Protocol |
| 59 | Kojima 2021^64^ | No primary closure |
| 60 | Kuncewitch 2019^65^ | Same data as Shen 2017^66^ |
| 61 | Kwon 2018^67^ | Within-subject experimental design |
| 62 | Lee 2017^68^ | Already included |
| 63 | Leitao 2020^69^ | Conference abstract of included study (Leitao 2021^70^) |
| 64 | Lopez 2022^71^ | Conference abstract of included study (Lopez-Lopez 2023^72^) |
| 65 | Low 2022^73^ | Protocol |
| 66 | Lozano-Balderas 2017^74^ | No primary closure |
| 67 | Lychagin 2020^75^ | Comparison not of interest |
| 68 | Martin 2019^76^ | Conference abstract of included study (O’neill 2020^77^) |
| 69 | Masters 2018^78^ | Same data as Masters 2021^79^ |
| 70 | Molina 2021^80^ | Conference abstract: no data available |
| 71 | Mondal 2022^81^ | Comparison not of interest |
| 72 | Mujahid 2020^82^ | Outcome not of interest |
| 73 | Muller-Sloof 2018^83^ | Already included |
| 74 | Murphy 2019^84^ | Already included |
| 75 | Myllykangas 2021^85^ | No randomization |
| 76 | NCT03815370, 2019^86^ | Protocol |
| 77 | NCT03816293, 2019^87^ | Protocol |
| 78 | NCT03820219, 2019^88^ | Protocol |
| 79 | NCT03871023, 2019^89^ | Protocol |
| 80 | NCT03886818, 2019^90^ | Protocol |
| 81 | NCT03900078, 2019^91^ | Protocol |
| 82 | NCT03905213, 2019^92^ | Protocol |
| 83 | NCT03935659, 2019^93^ | Protocol |
| 84 | NCT03948412, 2019^94^ | Protocol |
| 85 | NCT04003038, 2019^95^ | Protocol |
| 86 | NCT04039659, 2019^96^ | Protocol |
| 87 | NCT04063111, 2019^97^ | Protocol |
| 88 | NCT04088162, 2019^98^ | Protocol |
| 89 | NCT04110353, 2019^99^ | Protocol |
| 90 | NCT04174183, 2019^100^ | Protocol |
| 91 | NCT04265612, 2020^101^ | Protocol |
| 92 | NCT04434820, 2020^102^ | Protocol |
| 93 | NCT04453319, 2020^103^ | Protocol |
| 94 | NCT04455724, 2020^104^ | Protocol |
| 95 | NCT04496180, 2020^105^ | Protocol |
| 96 | NCT04520841, 2020^106^ | Protocol |
| 97 | NCT04539015, 2020^107^ | Protocol |
| 98 | NCT04584957, 2020^108^ | Protocol |
| 99 | Newman 2019, 2020^109^ | Already included |
| 100 | Ni 2020^110^ | No primary closure |
| 101 | Nip 2020^111^ | Conference abstract: no randomisation |
| 102 | Nordmeyer^112^ | No data available |
| 103 | Ozkan 2020^113^ | No randomisation |
| 104 | Paim / RBR-5c8y6v, 2019^114^ | Protocol |
| 105 | Pape 2021^115^ | Conference abstract: secondary analysis of included study (Tuuli 2020^116^) |
| 106 | Park 2019^117^ | No randomization |
| 107 | Pauser 2016^118^ | Comparison not of interest (drain in wound) |
| 108 | Png 2020^119^ | Same as Costa 2020^10^ |
| 109 | Pleger 2018^120^ | Within-subject experimental design |
| 110 | Rajabaleyan 2019^121^ | Conference abstract: no primary closure |
| 111 | Rezk 2019^122^ | Protocol |
| 112 | Sandy-Hodgetts 2017^123^ | Protocol |
| 113 | Sandy-Hodgetts 2020^124^ | Protocol |
| 114 | Sapci 2021^125^ | Conference abstract of included study (Sapci 2022^126^) |
| 115 | Schmid 2020^127^ | Within-subject experimental design |
| 116 | Schwartzmann 2021^128^ | No randomization |
| 117 | Seidel 2020^129^ | Comparison not of interest |
| 118 | Seidel 2020^130^ | No primary closure |
| 119 | Serra 2019^131^ | No randomization |
| 120 | Shim 2018^132^ | Duplicate |
| 121 | Stannard 2012^133^ | Within-subject experimental design |
| 122 | Sun 2019^134^ | Comparison not of interest |
| 123 | Svensson-Björk 2021^135^ | Outcome not of interest |
| 124 | Szmeja 2020^136^ | Conference abstract: no data available |
| 125 | Tanaydin 2018^137^ | Within-subject experimental design |
| 126 | Tanaydin 2018^138^ | Erratum |
| 127 | Venkatadass 2013^139^ | Retracted |
| 128 | Wang 2019^140^ | Comparison not of interest |
| 129 | Wilkin 2021^141^ | Protocol |
| 130 | Wilkin 2021^141^ | Protocol, duplicate |
| 131 | Wilkin 2022^142^ | Conference abstract: protocol |
| 132 | Yang 2020^143^ | Comparison not of interest |
| 133 | Yilmaz 2022^144^ | Protocol |
| 134 | Zhao 2020^145^ | No randomization |
| 135 | Zwanenburg 2020^146^ | Comment |
| **1.** Abesamis GM, Chopra S, Vickery K, Deva AK. A Comparative Trial of Incisional Negative-Pressure Wound Therapy in Abdominoplasty. *Plast Reconstr Surg Glob Open*. May 2019;7(5):e2141. doi:10.1097/GOX.0000000000002141.  **2.** Achten J, Vadher K, Bruce J, et al. Standard wound management versus negative-pressure wound therapy in the treatment of adult patients having surgical incisions for major trauma to the lower limb - a two-arm parallel group superiority randomised controlled trial: protocol for Wound Healing in Surgery for Trauma (WHIST). *Bmj Open*. Jun 2018;8(6)doi:ARTN e022115; 10.1136/bmjopen-2018-022115.  **3.** ACTRN12619000785101. Negative Pressure Wound Therapy to Reduce Incisional Wound Infections - A Randomised Control Trial. *https://trialsearchwhoint/Trial2aspx?TrialID=ACTRN12619000785101*. 2019.  **4.** Anderson V, Chaboyer W, Gillespie BM, Fenwick J. The use of negative pressure wound therapy dressing in obese women undergoing caesarean section:: a pilot study. *Evidence Based Midwifery*. 2014;12(1):23.  **5.** Chaboyer W, Anderson V, Webster J, Sneddon A, Thalib L, Gillespie BM. Negative Pressure Wound Therapy on Surgical Site Infections in Women Undergoing Elective Caesarean Sections: A Pilot RCT. *Healthcare (Basel)*. Sep 30 2014;2(4):417-28. doi:10.3390/healthcare2040417.  **6.** Biao Y, Shan W, Yan Z, L C. Treatment of chronic refractory wounds with negative pressure wound therapy and platelet-rich plasma: accelerating the re-epithelialization of wounds and increasing. *Chinese Journal of Tissue Engineering Research*. 23(26):4181.  **7.** Brennfleck FW, Linsenmeier L, Junger HHG, et al. Negative pressure wound therapy (NPWT) on closed incisions to prevent surgical site infection in high-risk patients in hepatopancreatobiliary surgery: study protocol for a randomized controlled trial—the NP-SSI trial. *Trials*. 2020 2020;21(1).  **8.** Brown S, Nixon J, Ransom M, et al. Multiple Interventions for Diabetic Foot Ulcer Treatment Trial (MIDFUT): study protocol for a randomised controlled trial. *BMJ open*. 2020 2020;10(4).  **9.** Campolier M, Knight R, Spoors L, Achten J, Costa M. Assessing the quality of data collection in clinic; lessons from the wound healing in surgical trauma (WHiST) RCT. *Trials*. 2019 2019;20.  **10.** Costa ML, Achten J, Knight R, et al. Effect of Incisional Negative Pressure Wound Therapy vs Standard Wound Dressing on Deep Surgical Site Infection After Surgery for Lower Limb Fractures Associated With Major Trauma: The WHIST Randomized Clinical Trial. *Jama*. 2020 2020;323(6):519-526.  **11.** Carrano FM, Maroli A, Carvello M, et al. Negative-pressure wound therapy after stoma reversal in colorectal surgery: a randomized controlled trial. *Bjs Open*. Nov 9 2021;5(6)doi:ARTN zrab116; 10.1093/bjsopen/zrab116  **12.** Chaboyer W, Ellwood D, Thalib L, et al. Incidence and predictors of surgical site infection in women who are obese and give birth by elective caesarean section: A secondary analysis. *Aust N Z J Obstet Gynaecol*. Apr 2022;62(2):234-240. doi:10.1111/ajo.13428  **13.** Gillespie BM, Webster J, Ellwood D, et al. Closed incision negative pressure wound therapy versus standard dressings in obese women undergoing caesarean section: multicentre parallel group randomised controlled trial. *BMJ*. May 5 2021;373:n893. doi:10.1136/bmj.n893  **14.** Chang EI. Discussion: Comparison between Negative-Pressure Fixation and Film Dressing in Wound Management after Tissue Expansion: A Randomized Controlled Trial. *Plastic and reconstructive surgery*. 2018 2018;142(1):42-43.  **15.** Chen SQ, Liu WC, Zhang ZZ, et al. [Application of closed negative pressure irrigation and suction device in the treatment of high perianal abscess]. *Zhonghua Wei Chang Wai Ke Za Zhi*. 2019 2019;22(4):364-369.  **16.** Chetter I, Arundel C, Martin BC, et al. Negative pressure wound therapy versus usual care for surgical wounds healing by secondary intention (SWHSI-2 trial): study protocol for a pragmatic, multicentre, cross surgical specialty, randomised controlled trial. *Trials*. Oct 25 2021;22(1):739. doi:10.1186/s13063-021-05662-2  **17.** ChiCTR1900022165. Topical continuous delivery of non-pressurised oxygen combined with negative pressure wound therapy for chronic wounds: A Randomized controlled trial. *https://trialsearchwhoint/Trial2aspx?TrialID=ChiCTR1900022165*. 2019;  **18.** ChiCTR2000034266. The effect and mechanism of negative pressure wound therapy on the survival rate and scar formation of modified Meek skin graft: a prospective randomized controlled study. *https://trialsearchwhoint/Trial2aspx?TrialID=ChiCTR2000034266*. 2020 2020;  **19.** Chu W, Liu S, Wang Y, Li J, Liu H. Compressed fixation combined with vacuum-assisted closure for treating acute injury of the heel fat pad. *Medical Science Monitor*. 2018 2018;24:9466-9472.  **20.** Clark JM, Rychlik S, Harris J, Seikaly H, Biron VL, O'Connell DA. Donor site morbidity following radial forearm free flap reconstruction with split thickness skin grafts using negative pressure wound therapy. *Le Journal d'oto-rhino-laryngologie et de chirurgie cervico-faciale [Journal of otolaryngology - head & neck surgery]*. 2019 2019;48(1):21.  **21.** Cocjin HGB, Jingco JKP, Tumaneng FDC, Coruña JMR. Wound-Healing Following Negative-Pressure Wound Therapy with Use of a Locally Developed AquaVac System as Compared with the Vacuum-Assisted Closure (VAC) System. *Journal of bone and joint surgery American volume*. 2019 2019;101(22):1990‐1998.  **22.** Cook R, Thomas V, Martin R. Negative pressure dressings are no better than standard dressings for open fractures. *BMJ (Online)*. 2019 2019;364  **23.** Costa ML, Achten J, Bruce J, et al. Negative-pressure wound therapy versus standard dressings for adults with an open lower limb fracture: The WOLLF RCT. *Health Technology Assessment*. 2018 2018;22(73):v-162.  **24.** Costa ML, Achten J, Bruce J, et al. Effect of negative pressure wound therapy vs standard wound management on 12-month disability among adults with severe open fracture of the lower limb the wollf randomized clinical trial. *JAMA - Journal of the American Medical Association*. 2018 2018;319(22):2280-2288.  **25.** Costa ML, Achten J, Knight R, et al. Negative-pressure wound therapy compared with standard dressings following surgical treatment of major trauma to the lower limb: the WHiST RCT. *Health Technol Assess*. 2020 2020;24(38):1-86.  **26.** CTRI/2019/05/019225. Clinical effectiveness of indigenous NPWT system Vacon for treatment of non healing wounds as compared to conventional wound therapy. 2019.  **27.** CTRI/2019/08/020895. To evaluate negative pressure dressings in decreasing surgical site infections after emergency laparotomy: a randomized controlled study. *https://trialsearchwhoint/Trial2aspx?TrialID=CTRI/2019/08/020895*. 2019.  **28.** CTRI/2019/09/021388. To compare number of infection between normal wounds dressing and wounds with machine dressing in open wounds after emergency operations of abdomen with pus: a comparison study. *https://trialsearchwhoint/Trial2aspx?TrialID=CTRI/2019/09/021388*. 2019 2019;  **29.** Dadras M, Ufton D, Sogorski A, et al. Closed-Incision Negative-Pressure Wound Therapy after Resection of Soft-Tissue Tumors Reduces Wound Complications: Results of a Randomized Trial. *Plastic and Reconstructive Surgery*. May 2022;149(5)doi:10.1097/Prs.0000000000009023  **30.** Darwisch A, Fajfrova Z. Negative pressure therapy after median sternotomy on closed incision: A randomized controlled study. *Thoracic and Cardiovascular Surgeon*. 2020 2020;68  **31.** Davis KE, La Fontaine J, Farrar D, et al. Randomized clinical study to compare negative pressure wound therapy with simultaneous saline irrigation and traditional negative pressure wound therapy for complex foot infections. *Wound Repair Regen*. 2020 2020;28(1):97-104.  **32.** Di Re AM, Wright D, Toh JWT, et al. Surgical wound infection prevention using topical negative pressure therapy on closed abdominal incisions – the ‘SWIPE IT’ randomized clinical trial. *Journal of Hospital Infection*. 2021 2021;110:76-83.  **33.** Dondossola D, Antonelli B, Rossi G. Vacuum-assisted wound closure and liver transplantation: new perspective and challenges. *Updates Surg*. Mar 2020;72(1):223-224. doi:10.1007/s13304-019-00693-6  **34.** Donlon NE, Boland PA, Kelly ME, et al. Prophylactic negative wound therapy in laparotomy wounds (PROPEL trial): randomized controlled trial. *Int J Colorectal Dis*. 2019 2019;34(11):2003-2010.  **35.** DRKS00015136. Negative pressure wound therapy (NPWT) on closed incisions to prevent surgical site infection in HPB-surgery. *https://trialsearchwhoint/Trial2aspx?TrialID=DRKS00015136*. 2019.  **36.** DRKS00021494. Single use negative pressure wound therapy system (Prevena ™) compared to standard wound care after spinal surgery. *https://trialsearchwhoint/Trial2aspx?TrialID=DRKS00021494*. 2020.  **37.** Engelhardt M, Rashad NA, Willy C, et al. Closed-incision negative pressure therapy to reduce groin wound infections in vascular surgery: a randomised controlled trial. *International Wound Journal*. 2018 2018;15(3):327-332.  **38.** Fang CL, Changchien CH, Chen MS, Hsu CH, Tsai CB. Closed incision negative pressure therapy following abdominoplasty after breast reconstruction with deep inferior epigastric perforator flaps. *Int Wound J*. 2020 2020;17(2):326-331.  **39.** Fernandes U, Marçal A, Pereira R, et al. The Impact of Closed Incision Negative Pressure Therapy on Postoperative Oncologic Breast Surgery Outcomes. *European Journal of Surgical Oncology*. 2021 2021;47(2):e45.  **40.** Ferrando P, Castellano I, Folli S, et al. A national multicenter randomized controlled trial to evaluate Closed Incision Negative Pressure Therapy in oncological breast surgery. *European Journal of Surgical Oncology*. 2021 2021;47(2):e16-e17.  **41.** Fogacci T, Cattin F, Samorani D. The negative pressure therapy with PICO as a prevention of surgical site infection in high risk patients undergoing breast surgery. *Annals of Oncology*. 2019 2019;30:iii42.  **42**. Fogacci T, Cattin F, Semprini G, Frisoni G, Fabiocchi L, Samorani D. The negative pressure therapy with PICO as a prevention of surgical site infection in high-risk patients undergoing breast surgery. *Breast J*. May 2020;26(5):1071-1073. doi:10.1111/tbj.13659  **43.** Galiano RD, Hudson D, Shin J, et al. Incisional Negative Pressure Wound Therapy for Prevention of Wound Healing Complications Following Reduction Mammaplasty. *Plast Reconstr Surg Glob Open*. Jan 2018;6(1):e1560. doi:10.1097/GOX.0000000000001560  **44.** Gombert A, Babilon M, Barbati ME, et al. Closed Incision Negative Pressure Therapy Reduces Surgical Site Infections in Vascular Surgery: A Prospective Randomised Trial (AIMS Trial). *Eur J Vasc Endovasc Surg*. Sep 2018;56(3):442-448. doi:10.1016/j.ejvs.2018.05.018  **45.** Gombert A, Babilon M, Barbati M, et al. Closed-incision Negative-pressure Therapy Reduces Surgical Site Infections in Vascular Surgery: A Prospective Randomised Controlled Trial (Aims Trial). *European Journal of Vascular and Endovascular Surgery*. 2019 2019;58(6):e359.  **46.** Gombert A, Babilon M, Barbati M, et al. Correction: Closed incisional negative pressure therapy may reduce surgical site infection rate following endophlebectomy with complementary polytetrafluoroethylene arteriovenous fistula of the common femoral vein (Journal of Vascular Surgery: Venous and Lymphatic Disorders (2020) 8(1) (89–94), (S2213333X1930438X), (10.1016/j.jvsv.2019.08.010)). *Journal of Vascular Surgery: Venous and Lymphatic Disorders*. 2020 2020;8(2):339.  **47.** Gonzalez MG, Elisa Barske M, Kjellsson KB, Saboda K, Hill MG. Topical negative pressure wound therapy to prevent wound complications following cesarean delivery in high risk obstetric patients. *Reproductive Sciences*. 2020 2020;27(1):136A-137A.  **48.** Haddad T, Bocchese S, Staley C, et al. Preliminary Analysis of Negative Pressure to Prevent Lower Extremity Amputation Wound Complications: Pilot Data From a Randomized Clinical Trial. *Journal of Vascular Surgery*. Sep 2021;74(3):E153-E153.  **49.** Halama D, Dreilich R, Lethaus B, Bartella A, Pausch NC. Donor-site morbidity after harvesting of radial forearm free flaps-comparison of vacuum-assisted closure with conventional wound care: A randomized controlled trial. *J Craniomaxillofac Surg*. 2019 2019;47(12):1980-1985.  **50.** Hasselmann J, Björk J, Svensson-Björk R, Acosta S. Inguinal Vascular Surgical Wound Protection by Incisional Negative Pressure Wound Therapy – A Randomized Controlled Trial – INVIPS Trial. *European Journal of Vascular and Endovascular Surgery*. 2019 2019;58(6):e726-e727.  **51.** Hasselmann J, Björk J, Svensson-Björk R, Butt T, Acosta S. Proposed Classification of Incision Complications: Analysis of a Prospective Study on Elective Open Lower-Limb Revascularization. *Surg Infect (Larchmt)*. 2020 2020;21(4):384-390.  **52.** Hasselmann J, Bjork J, Svensson-Bjork R, Acosta S. Inguinal Vascular Surgical Wound Protection by Incisional Negative Pressure Wound Therapy: A Randomized Controlled Trial-INVIPS Trial. *Ann Surg*. Jan 2020;271(1):48-53. doi:10.1097/SLA.0000000000003364.  **53.** Howell RD, Hadley S, Strauss E, Pelham FR. Blister formation with negative pressure dressings after total knee arthroplasty. *Current Orthopaedic Practice*. 2011;22(2):176-179.  **54.** Hyldig N, Vinter CA, Kruse M, et al. Prophylactic incisional negative pressure wound therapy reduces the risk of surgical site infection after caesarean section in obese women: a pragmatic randomised clinical trial. *BJOG*. Apr 2019;126(5):628-635. doi:10.1111/1471-0528.15413.  **55.** Hyldig N, Joergensen JS, Wu C, et al. Cost-effectiveness of incisional negative pressure wound therapy compared with standard care after caesarean section in obese women: a trial-based economic evaluation. *BJOG*. 2019 2019;126(5):619-627.  **56.** Jaimes HG, B. Performance, safety, and efficacy of a single-use negative pressure wound therapy system for surgically closed incision sites and skin grafts: A prospective multi-center follow-up study. 2020.  **57.** Javed AA, Teinor J, Wright M, et al. Negative Pressure Wound Therapy for Surgical-site Infections: A Randomized Trial. *Ann Surg*. 2019 2019;269(6):1034-1040.  **58.** Jenkins S, Komber M, Mattam K, Briffa N. Negative pressure wound therapy in patients with diabetes undergoing left internal thoracic artery harvest: A randomized control trial. *J Thorac Cardiovasc Surg*. Apr 9 2022;doi:10.1016/j.jtcvs.2022.01.060  **59.** Jørgensen MG, Toyserkani NM, Hyldig N, et al. Prevention of seroma following inguinal lymph node dissection with prophylactic, incisional, negative-pressure wound therapy (SEROMA trial): study protocol for a randomized controlled trial [published correction appears in Trials. 2018 Oct 19;19(1):570]. *Trials*. 2018;19(1):441. Published 2018 Aug 15. doi:10.1186/s13063-018-2757-6  **60.** Should negative pressure wound therapy be used at the time of caesarean in obese women? *BJOG: An International Journal of Obstetrics and Gynaecology*. 2019 2019;126(5):636.  **61.** KCT0004063. The effectiveness of negative pressure wound dressing for the wound healing after stoma closure: An prospective, open-label, randomized control study. *https://trialsearchwhoint/Trial2aspx?TrialID=KCT0004063*. 2019.  **62.** Kim S, Kang SI. The effectiveness of negative-pressure wound therapy for wound healing after stoma reversal: a randomised control study (SR-PICO study). *Trials*. 2020 2020;21(1):24.  **63.** Knight R, Spoors LM, Costa ML, Dutton SJ. Wound Healing In Surgery for Trauma (WHIST): statistical analysis plan for a randomised controlled trial comparing standard wound management with negative pressure wound therapy. *Trials*. 2019 2019;20(1):186.  **64.** Kojima K, Goto M, Nagashima Y, et al. Effectiveness of negative pressure wound therapy for the wound of ileostomy closure: a multicenter, phase II randomized controlled trial. *Bmc Surgery*. Dec 28 2021;21(1)doi:ARTN 442 10.1186/s12893-021-01446-2  **65.** Kuncewitch MP, Blackham AU, Clark CJ, et al. Effect of Negative Pressure Wound Therapy on Wound Complications Post-Pancreatectomy. *Am Surg*. 2019 2019;85(1):1-7.  **66.** Shen P, Blackham AU, Lewis S, et al. Phase II Randomized Trial of Negative-Pressure Wound Therapy to Decrease Surgical Site Infection in Patients Undergoing Laparotomy for Gastrointestinal, Pancreatic, and Peritoneal Surface Malignancies. *J Am Coll Surg*. Apr 2017;224(4):726-737. doi:10.1016/j.jamcollsurg.2016.12.028  **67.** Kwon J, Staley C, McCullough M, et al. A randomized clinical trial evaluating negative pressure therapy to decrease vascular groin incision complications. *J Vasc Surg*. Dec 2018;68(6):1744-1752. doi:10.1016/j.jvs.2018.05.224  **68.** Lee K, Murphy PB, Ingves MV, et al. Randomized clinical trial of negative pressure wound therapy for high-risk groin wounds in lower extremity revascularization. *Journal of Vascular Surgery*. 2017 2017;66(6):1814-1819.  **69.** Leitao MM, Zhou Q, Schiavone MB, et al. A phase 3 randomized controlled trial of preventive negative pressure wound therapy in postoperative incision management. *Gynecologic Oncology*. 2020 2020;159:53.  **70.** Leitao MM, Jr., Zhou QC, Schiavone MB, et al. Prophylactic Negative Pressure Wound Therapy After Laparotomy for Gynecologic Surgery: A Randomized Controlled Trial. *Obstet Gynecol*. Feb 1 2021;137(2):334-341. doi:10.1097/AOG.0000000000004243  **71.** Lopez VL, Martinez-Alarcon L, Hiciano-Guillermo A, et al. Postoperative negative-pressure incision therapy following liver transplant (ponilitrans study): a randomized controlled trial. *Transplantation*. Aug 2022;106(8s):101-101.  **72.** Lopez-Lopez V, Hiciano-Guillermo A, Martinez-Alarcon L, et al. Postoperative negative-pressure incision therapy after liver transplant (PONILITRANS study): A randomized controlled trial. *Surgery*. Apr 2023;173(4):1072-1078. doi:10.1016/j.surg.2022.11.011  **73.** Low EZ, Nugent TS, O'Sullivan NJ, et al. Application of PREVENA (Surgical Incision Protection System) in reducing surgical site infections following reversal of ileostomy or colostomy: the PRIC study protocol. *International Journal of Colorectal Disease*. May 2022;37(5):1215-1221. doi:10.1007/s00384-022-04153-3  **74.** Lozano-Balderas G, Ruiz-Velasco-Santacruz A, Diaz-Elizondo JA, Gomez-Navarro JA, Flores-Villalba E. Surgical Site Infection Rate Drops to 0% Using a Vacuum-Assisted Closure in Contaminated/Dirty Infected Laparotomy Wounds. *Am Surg*. May 1 2017;83(5):512-514.  **75.** Lychagin AV, Rosenberg N, Gritsyuk AA. Evaluation of the potential complications of surgical wound drainage in primary total hip arthroplasty: a prospective controlled double-blind study. *Hip Int*. 2020 2020:1120700020941749.  **76.** Martin RCG, O'Neill CH. Negative-pressure therapy for hepatectomy and pancreatectomy: A randomized trial for surgical site infection prevention. *HPB*. 2019 2019;21:S26-S27.  **77.** O'Neill CH, Martin RCG, 2nd. Negative-pressure wound therapy does not reduce superficial SSI in pancreatectomy and hepatectomy procedures. *J Surg Oncol*. Sep 2020;122(3):480-486. doi:10.1002/jso.25980  **78.** Masters JPM, Achten J, Cook J, Dritsaki M, Sansom L, Costa ML. Randomised controlled feasibility trial of standard wound management versus negative-pressure wound therapy in the treatment of adult patients having surgical incisions for hip fractures. *BMJ Open*. 2018 2018;8(4)  **79.** Masters J, Cook J, Achten J, Costa ML, Group WS. A feasibility study of standard dressings versus negative-pressure wound therapy in the treatment of adult patients having surgical incisions for hip fractures: the WHISH randomized controlled trial. *Bone Joint J*. Apr 2021;103-B(4):755-761. doi:10.1302/0301-620X.103B4.BJJ-2020-1603.R1  **80.** Molina AC, Pla MJ. Negative Pressure Therapy in the Prevention of Surgical Wound Complications in Breast Oncoplastic Surgery. A Prospective Randomized Study. *International Journal of Gynecological Cancer*. Mar 2021;31:A372-A372. doi:10.1136/ijgc-2021-ESGO.657  **81.** Mondal A, Ali MS, Galidevara I, Arumugam M. Effect of Incisional Negative Pressure Wound Therapy Following Incisional Hernia Repair-A Randomised Controlled Trial. *JCDR* Feb 2022;16(2):1-4. doi: 10.7860/JCDR/2022/51153.15955.  **82.** Mujahid AM, Khalid FA, Ali N, Sajjad Y, Khan H, Tarar MN. Vacuum-assisted Closure in Integration of Skin Graft Over Scalp Wounds: A Randomised Control Trial. *J Coll Physicians Surg Pak*. 2020 2020;30(2):163-167.  **83.** Muller-Sloof E, de Laat HEW, Hummelink SLM, Peters JWB, Ulrich DJO. The effect of postoperative closed incision negative pressure therapy on the incidence of donor site wound dehiscence in breast reconstruction patients: DEhiscence PREvention Study (DEPRES), pilot randomized controlled trial. *Journal of Tissue Viability*. 2018 2018;27(4):262-266.  **84.** Murphy PB, Knowles S, Chadi SA, et al. Negative Pressure Wound Therapy Use to Decrease Surgical Nosocomial Events in Colorectal Resections (NEPTUNE): A Randomized Controlled Trial. *Ann Surg*. 2019 2019;270(1):38-42.  **85.** Myllykangas HM, Halonen J, Husso A, Vaananen H, Berg LT. Does Incisional Negative Pressure Wound Therapy Prevent Sternal Wound Infections? *Thorac Cardiovasc Surg*. Jan 2022;70(1):65-71. doi:10.1055/s-0041-1731767  **86.** NCT03815370. A Non-Traumatic Binder for Temporary Abdominal Wall Closure. *https://clinicaltrialsgov/show/NCT03815370*. 2019.  **87.** NCT03816293. SUpPress SSI - Single Use Negative Pressure Wound Therapy (NPWT) to Reduce Surgical Site Infections. *https://clinicaltrialsgov/show/NCT03816293*. 2019 2019.  **88.** NCT03820219. Incisional Negative Pressure Wound Therapy in Patients Undergoing Spine Surgery. *https://clinicaltrialsgov/show/NCT03820219*. 2019.  **89.** NCT03871023. Prophylactic Negative Wound Therapy in Laparotomy Wounds. *https://clinicaltrialsgov/show/NCT03871023*. 2019.  **90.** NCT03886818. Efficacy of Negative Pressure Wound Therapy After Total Ankle Arthroplasty. *https://clinicaltrialsgov/show/NCT03886818*. 2019.  **91.** NCT03900078. Incisional Negative Pressure Wound Therapy for Resection of Soft Tissue Tumors. *https://clinicaltrialsgov/show/NCT03900078*. 2019.  **92.** NCT03905213. Prevention of Surgical Wound Infection. *https://clinicaltrialsgov/show/NCT03905213*. 2019.  **93.** NCT03935659. Negative Pressure Wound Therapy for Surgical Site Infection Prevention in Common Femoral Artery Exposure. *https://clinicaltrialsgov/show/NCT03935659*. 2019.  **94.** NCT03948412. Negative Pressure Wound Therapy (PREVENA) Versus Standard Dressings for Incision Management After Renal Transplant. *https://clinicaltrialsgov/show/NCT03948412*. 2019.  **95.** NCT04003038. Negative Pressure Wound Therapy in Healing Abdominal Incision in Obese Patients Undergoing Breast Reconstruction Surgery. *https://clinicaltrialsgov/show/NCT04003038*. 2019.  **96.** NCT04039659. POstoperative Negative-pressure Incision Therapy Following LIver TRANSplant: a Randomized Controlled Trial. *https://clinicaltrialsgov/show/NCT04039659*. 2019.  **97.** NCT04063111. Role of Vacuum in Open Fracture Tibia Grade III Type B. *https://clinicaltrialsgov/show/NCT04063111*. 2019.  **98.** NCT04088162. The Use of Post-operative NPWT Dressing in the Prevention of Infectious Complications After Ostomy Reversal Surgery. *https://clinicaltrialsgov/show/NCT04088162*. 2019.  **99.** NCT04110353. Prophylactic Closed Incision Negative Pressure Wound Therapy on Abdominal Wounds - Clinical and Economic Perspectives. *https://clinicaltrialsgov/show/NCT04110353*. 2019.  **100.** NCT04174183. Evaluation of the Effectiveness of a Closed-incision Negative-pressure Therapy (Prevena®) on Bilateral Groin Incision. *https://clinicaltrialsgov/show/NCT04174183*. 2019.  **101.** NCT04265612. Effect of the Negative Pressure Therapy Dressing Compared With Hydrogel Dressing. *https://clinicaltrialsgov/show/NCT04265612*. 2020.  **102.** NCT04434820. External Negative Pressure Dressing System vs. Traditional Wound Dressing for Cesarean Section Incision in Obese Women. *https://clinicaltrialsgov/show/NCT04434820*. 2020.  **103.** NCT04453319. Efficacy of Negative Pressure Wound Closure Therapy by PICO System in Prevention of Complications of Femoral Artery Exposure. *https://clinicaltrialsgov/show/NCT04453319*. 2020.  **104.** NCT04455724. Negative Pressure Incisional Wound Therapy for High-risk Ventral Hernia Repair: a Randomized Controlled Trial. *https://clinicaltrialsgov/show/NCT04455724*. 2020.  **105.** NCT04496180. Prevena to Prevent Surgical Site Infection After Emergency Abdominal Laparotomy. *https://clinicaltrialsgov/show/NCT04496180*. 2020.  **106.** NCT04520841. Clinical Trial Comparing Negative Pressure Wound Therapy and Standard Dry Dressings. *https://clinicaltrialsgov/show/NCT04520841*. 2020.  **107.** NCT04539015. Assess the Efficacy of Prevena Plus vs SOC to Closed Incision in Pts Undergoing CAWR and Other Laparotomy Procedures. *https://clinicaltrialsgov/show/NCT04539015*. 2020.  **108.** NCT04584957. Prophylactic Negative Pressure Wound Therapy (VAC) in Gynecologic Oncology (G.O.). *https://clinicaltrialsgov/show/NCT04584957*. 2020.  **109.** Newman JM, Siqueira MBP, Klika AK, Molloy RM, Barsoum WK, Higuera CA. Use of Closed Incisional Negative Pressure Wound Therapy After Revision Total Hip and Knee Arthroplasty in Patients at High Risk for Infection: A Prospective, Randomized Clinical Trial. *J Arthroplasty*. 2019 2019;34(3):554-559.e1.  **110.** Ni Z, Sun J, Qi S. Therapeutic Effect of Topical Negative Pressure Therapy/Vacuum-Associated Closure Therapy on Cephalic Facial Skin Abscess. *Surg Infect (Larchmt)*. 2020 2020;21(8):722-725.  **111.** Nip L, Fatayer H, Rusius V, Bramley M. Surgical Site Infection (SSI) and seroma rates in oncoplastic breast patients using negative pressure wound dressings. *European Journal of Surgical Oncology*. 2020 2020;46(6):e34-e35.  **112.** Nordmeyer M, Pauser J, Biber R, et al. Negative pressure wound therapy for seroma prevention and surgical incision treatment in spinal fracture care. *Int Wound J*. 2016;13(6):1176-1179. doi:10.1111/iwj.12436.  **113.** Ozkan B, Markal Ertas N, Bali U, Uysal CA. Clinical Experiences with Closed Incisional Negative Pressure Wound Treatment on Various Anatomic Locations. *Cureus*. 2020 2020;12(6):e8849.  **114.** RBR-5c8y6v. The Wound action effects of a Simple Suction Dressing. *http://wwwwhoint/trialsearch/Trial2aspx?TrialID=RBR-5c8y6v*. 2019 2019;  **115.** Pape K, Tuuli MG, Neal CM, et al. Predictors of surgical-site infection after cesarean delivery in obese women receiving evidence-based preventive measures. *American Journal of Obstetrics and Gynecology*. 2021 2021;224(2):S652-S653.  **116.** Tuuli MG, Liu J, Tita ATN, et al. Effect of Prophylactic Negative Pressure Wound Therapy vs Standard Wound Dressing on Surgical-Site Infection in Obese Women After Cesarean Delivery: A Randomized Clinical Trial. *JAMA*. Sep 22 2020;324(12):1180-1189. doi:10.1001/jama.2020.13361.  **117.** Park KU, Clemens MW, Lange CE, Bridges CA, Checka CM. Novel Use of Incisional Negative Pressure Wound Therapy for Management of High-risk Breast Incisions. *Ann Surg*. 2019 2019;270(6):e73-e74.  **118.** Pauser J, Nordmeyer M, Biber R, et al. Incisional negative pressure wound therapy after hemiarthroplasty for femoral neck fractures - reduction of wound complications. *Int Wound J*. 2016;13(5):663-667. doi:10.1111/iwj.12344.  **119.** Png ME, Madan JJ, Dritsaki M, et al. Cost-utility analysis of standard dressing compared with incisional negative-pressure wound therapy among patients with closed surgical wounds following major trauma to the lower limb. *Bone Joint J*. 2020 2020;102(8):1072-1081.  **120.** Pleger SP, Nink N, Elzien M, Kunold A, Koshty A, Boning A. Reduction of groin wound complications in vascular surgery patients using closed incision negative pressure therapy (ciNPT): a prospective, randomised, single-institution study. *Int Wound J*. Feb 2018;15(1):75-83. doi:10.1111/iwj.12836  **121.** Rajabaleyan P. Vacuum assisted closure versus on-demand re-laparotomy in patients with faecal or diffuse peritonitis: a multicenter randomized controlled trial (VACOR). *Colorectal disease*. 2019 2019;21:17‐.  **122.** Rezk F, Åstrand H, Acosta S. Incisional negative pressure wound therapy for the prevention of surgical site infection after open lower limb revascularization – Rationale and design of a multi-center randomized controlled trial. *Contemporary Clinical Trials Communications*. 2019 2019;16  **123.** Sandy-Hodgetts K, Leslie GD, Parsons R, Zeps N, Carville K. Prevention of postsurgical wound dehiscence after abdominal surgery with NPWT: a multicentre randomised controlled trial protocol. *Journal of wound care*. 2017 2017;26:S23-S26.  **124.** Sandy-Hodgetts K, Parsons R, Norman R, Fear MW, Wood FM, White SW. Effectiveness of negative pressure wound therapy in the prevention of surgical wound complications in the cesarean section at-risk population: a parallel group randomised multicentre trial-the CYGNUS protocol. *BMJ Open*. 2020 2020;10(10):e035727.  **125.** Sapci I, Hull T, Ashburn JH, et al. The American Society of Colon and Rectal Surgeons 2021 Annual Scientific Meeting Abstracts. *Diseases of the Colon & Rectum*. 2021;64(5):e109-e364. doi:10.1097/dcr.0000000000002029  **126.** Sapci I, Hull T, Ashburn JH, et al. Effect of Incisional Negative Pressure Wound Therapy on Surgical Site Infections in High Risk Re-Operative Colorectal Surgery: A Randomized Controlled Trial. *Dis Colon Rectum*. May 2021;64(5)  **127.** Schmid SC, Seitz AK, Haller B, et al. Final results of the PräVAC trial: prevention of wound complications following inguinal lymph node dissection in patients with penile cancer using epidermal vacuum-assisted wound closure. *World J Urol*. 2021 2021;39(2):613-620.  **128.** Schwartzmann E, Sy M, Sharma M, Mankowski B, Jemielity M, Perek B. Negative pressure wound therapy for surgical site infection after sternotomy and its role in preparing the wound for reconstruction. *Kardiochir Torakochirurgia Pol*. Sep 2021;18(3):190-191. doi:10.5114/kitp.2021.109414  **129.** Seidel D, Storck M, Lawall H, et al. Negative pressure wound therapy compared with standard moist wound care on diabetic foot ulcers in real-life clinical practice: results of the German DiaFu-RCT. *BMJ open*. 2020 2020;10(3):e026345.  **130.** Seidel D, Diedrich S, Herrle F, et al. Negative Pressure Wound Therapy vs Conventional Wound Treatment in Subcutaneous Abdominal Wound Healing Impairment: The SAWHI Randomized Clinical Trial. *JAMA Surg*. 2020 2020;155(6):469-478.  **131.** Serra F, Sergi W, Spatafora F, et al. Negative pressure wound therapy (NPWT) after cytoreductive surgery (CRS) and intraperitoneal chemotherapy (HIPEC) for peritoneal surface malignancies: preliminary report. *G Chir*. 2019 2019;40(6):578-582.  **132.** Shim HS, Choi JS, Kim SW. A Role for Postoperative Negative Pressure Wound Therapy in Multitissue Hand Injuries. *Biomed Res Int*. 2018;2018:3629643. doi:10.1155/2018/3629643  **133.** Stannard JP, Volgas DA, McGwin G, 3rd, et al. Incisional negative pressure wound therapy after high-risk lower extremity fractures. *J Orthop Trauma*. Jan 2012;26(1):37-42. doi:10.1097/BOT.0b013e318216b1e5  **134.** Sun W, Gao JH, Zhu LG, et al. Compression therapy following posterior lumbar interbody fusion: a prospective, randomized, clinical study. *BMC Surg*. 2019 2019;19(1):161.  **135.** Svensson-Björk R, Saha S, Acosta S, et al. Cost-effectiveness analysis of negative pressure wound therapy dressings after open inguinal vascular surgery – The randomised INVIPS-Trial. *Journal of Tissue Viability*. 2021 2021;30(1):95-101.  **136.** Szmeja J, Borejsza-Wysocki M, Bobkiewicz A, Krokowicz L, Banasiewicz T, Szmyt K. The Comparison of Quality of Life in Patients with Pilonidal Sinus Disease. Negative Pressure Wound Therapy Versus Standard Wound Dressings - A Randomized Pilot Study. *Gastroenterology*. 2020 2020;158(6):S‐1601‐.  **137.** Tanaydin V, Beugels J, Andriessen A, Sawor JH, van der Hulst RRWJ. Randomized Controlled Study Comparing Disposable Negative-Pressure Wound Therapy with Standard Care in Bilateral Breast Reduction Mammoplasty Evaluating Surgical Site Complications and Scar Quality. *Aesthetic plastic surgery*. 2018 2018;42(4):927-935.  **138.** Tanaydin V, Beugels J, Andriessen A, Sawor JH, van der Hulst RRWJ. Erratum: Correction to: Randomized Controlled Study Comparing Disposable Negative-Pressure Wound Therapy with Standard Care in Bilateral Breast Reduction Mammoplasty Evaluating Surgical Site Complications and Scar Quality (Aesthetic plastic surgery (2018) 42 4 (927-935)). *Aesthetic plastic surgery*. 2018 2018;42(4):1176.  **139.** Venkatadass K, Bittersohl B, Fornari ED, Bomar JD, Hosalkar H. Erratum: Does incisional wound VAC after major Hip surgery in obese pediatric patients reduce wound infection and scar formation? A pilot study pediatrics (Clinical Orthopaedics and Related Research). *Clinical Orthopaedics and Related Research*. 2013 2013;471(8):2730.  **140.** Wang T, Li X, Fan L, et al. Negative pressure wound therapy promoted wound healing by suppressing inflammation via down-regulating MAPK-JNK signaling pathway in diabetic foot patients. *Diabetes Res Clin Pract*. 2019 2019;150:81-89.  **141.** Collaborative SUNRRISE Study Group on behalf of the Northwest Research Collaborative and the West Midlands Research Collaborative. An international pragmatic randomised controlled trial to compare a single use negative pressure dressing versus a surgeon's preference of dressing to reduce the incidence of surgical site infection following emergency laparotomy: the SUNRRISE Trial Protocol. *Colorectal Dis*. Dec 4 2020;doi:10.1111/codi.15474  **142.** Wilkin R. The SUNRRISE Trial – Single Use Negative pressure dressing for Reduction In Surgical site infection following Emergency laparotomy. 2022;  **143.** Yang ML, Zhou XJ, Zhu YG, et al. [Clinical efficacy and influencing factors of different modes of continuous negative pressure wound therapy on venous ulcer wounds of lower limbs]. *Zhonghua Shao Shang Za Zhi*. 2020 2020;36(12):1149-1158.  **144.** Yilmaz M, Thorn A, Sorensen MS, Jensen CL, Petersen MM. Effect of negative pressure wound therapy after surgical removal of deep-seated high-malignant soft tissue sarcomas of the extremities and trunk wall-study protocol for a randomized controlled trial. *Trials*.Jun 18 2022;23(1)doi:ARTN 507; 10.1186/s13063-022-06468-6  **145.** Zhao N, Liu Y, Yue J, et al. Negative pressure drainage-assisted irrigation for maxillofacial space infection. *Oral Dis*. 2020 2020;26(7):1586-1591.  **146.** Zwanenburg PR, Timmer AS, Boermeester MA. Incisional Negative Pressure Wound Therapy After Surgery for Major Trauma-Related Fractures. *Jama*. 2020 2020;323(22):2343-2344. | | |

# **Appendix 4. Study characteristics**

| **Study** | **N total** | **Procedure** | **Wound class** | **SAP** | **iNPWT device** | **mmHg** | **Min. days** | **Control dressings** | **Industry involvement in design** | **ROB** |  |
| --- | --- | --- | --- | --- | --- | --- | --- | --- | --- | --- | --- |
| Andrianello 2021 | 100 | Pancreatectomy | II | Yes | PICO | -80 * | 7 | GBDs | No | Some concerns |  |
| Arellano 2021 | 148 | Colorectal surgery | II-III | Yes | Prevena | -125 * | 7 | GBDs | Yes | Some concerns |  |
| Bertges 2021 | 252 | Open femoral vascular surgery | I / IV | Yes | Prevena | -125 * | 5 | GBDs | Yes | Some concerns |  |
| Borejsza-Wysocki 2021 | 30 | Stoma reversal surgery | III | Yes | PICO | -80 | 7 | GBDs | Not reported | Some concerns |  |
| Bueno-Lledó 2021 | 146 | Hernia repair | I | Yes | PICOn | -80 | 7 | GBDs | No | Some concerns |  |
| Canton 2020 | 65 | Lower extremity fracture surgery | I | Yes | PICO | -80 | 7 | GBDs | No | High |  |
| Chaboyer 2014 | 87 | Caesarean section | II | Yes | PICO | -80 * | 4 | HBDs | No | Low |  |
| Cooper 2022 | 120 | Total hip arthroplasty | I | NR | Prevena | -125 * | 7 | HBDs | No | Some concerns |  |
| Costa 2020 | 1629 | Lower extremity fracture surgery | I | NR | PICO | -80 * | 7 | GBDs | No | Some concerns |  |
| Crist 2017 | 66 | Acetabular fracture surgery | I | Yes | VAC | -125 | 2 | GBDs | No | Some concerns |  |
| Di Re 2021 | 127 | Open abdominal surgery | I-IV | Yes | Prevena | -125 | 5 | GBDs or HBDs | No | Some concerns |  |
| Engelhardt 2018 | 132 | Open femoral vascular surgery | I | Yes | Prevena | -125 | 5 | GBDs | Not reported | High |  |
| Flynn 2020 | 201 | Open abdominal surgery | II-III | Yes | PICO | -80 * | 7 | NR | No | Some concerns |  |
| Fogacci 2019 | 100 | Breast surgery | I | NR | PICO | -80 | 7 | GBDs | Not reported | Some concerns |  |
| Gabriele 2021 | 52 | Oncological orthopedic surgery | I | Yes | PICO | -80 | 7 | ‘Standard’ | Not reported | High |  |
| Garg 2021 | 50 | Emergency abdominal laparotomy | I-IV? | NR | NR | -75 | 3 | GBDs | Not reported | Some concerns |  |
| Gillespie 2015 | 70 | Primary hip arthroplasty | I | Yes | PICO | -80 | 5§ | HBDs | No | Low |  |
| Gillespie 2021 | 2035 | Caesarean section | II | Yes | PICO | -80 | 5 | GBDs or GBDs | No | Low |  |
| Gök 2019 | 40 | General surgery | II-IV | NR | Prevena | -125 * | 7 | ‘Standard’ | Not reported | High |  |
| Gombert 2018 | 204 | Open femoral vascular surgery | I | Yes | Prevena | -125 | 5 | GBDs | No | Low |  |
| Gunatilake 2017 | 82 | Caesarean section | I-II | Yes | Prevena | -125 | 5 | GBDs or HBDs | Yes | High |  |
| Hasselmann 2020 | 154 | Inguinal vascular surgery | I | Yes | PICO | -80 | NR | GBDs | No | High |  |
| Higuera-Rueda 2021 | 294 | Knee arthroplasty | I / IV | NR | Prevena | -125 | 5 | HBDs with silver | Yes | Some concerns |  |
| Hussamy 2019 | 241 | Caesarean section | II | Yes | Prevena | -125 | 2 | GBDs | No | Some concerns |  |
| Hyldig 2018 | 876 | Caesarean section | II | Yes | PICO | -80 * | 5 | GBDs | No | Some concerns |  |
| Javed 2019 | 123 | Open pancreaticoduodenectomy | II | Yes | Prevena | -125 | 5 | GBDs | No | Low |  |
| Kacmaz 2022 | 56 | Colorectal cancer surgery | II-III | Yes | PICO | -80 | 7 | GBDs | No | Some concerns |  |
| Karlakki 2016 | 209 | Hip and knee arthroplasty | I | Yes | PICO | -80 * | 7 | GBDs or HBDs | Yes | Some concerns |  |
| Keeney 2019 | 398 | Hip and knee arthroplasty | I | NR | PICO | -80 | 7 | GBDs | Yes | High |  |
| Lee 2017, cardiac | 64 | Saphenous vein harvest for CABG | I | NR | Prevena | -125 | 7§ | GBDs | Yes | Low |  |
| Lee 2017, vascular | 102 | Open femoral vascular surgery | I | NR | Prevena | -125 * | 8§ | GBDs | No | Low |  |
| Leitao 2021 | 584 | Open gynecologic surgery | I-IV | Yes | Prevena | -125 | 7§ | ‘Standard’ | Yes | Some concerns |  |
| Leon 2016 | 81 | Open colorectal surgery | II | NR | NR | NR | NR | ‘Standard’ | Not reported | High |  |
| Li 2017 | 71 | Open abdominal surgery | I-II | Yes | VSD | -125 | 3 | GBDs | No | Low |  |
| Lopez 2023 | 120 | Liver transplant surgery | II | Yes | PICO | -80 | 5 | GBDs | No | Some concerns |  |
| Masden 2012 | 81 | Lower extremity or abdominal wound closure | I | NR | VAC | -125 | 3 | Silicone gauze + silver dressing | Not reported | High |  |
| Masters 2021 | 465 | Hip fracture surgery | I | Yes | PICO | -80 | NR | ‘Standard’ | No | Some concerns |  |
| Muller-Sloof 2018 | 51 | DIEAP or PAP donor-site closure | I | Yes | Prevena | -125 | 5 | GBDs | No | Some concerns |  |
| Muller-Sloof 2022 | 80 | DIEP flap breast reconstruction | I | Yes | Prevena | -125 | 5 | NR | No | Some concerns |  |
| Murphy 2019 | 300 | Colorectal surgery | II | Yes | Prevena | -125 | 5 | GBDs | No | Some concerns |  |
| Newman 2019 | 160 | Hip and knee arthroplasty | I | Yes | Prevena | -125 * | 2 | GBDs with silver | Yes | Some concerns |  |
| O'Leary 2017 | 49 | Open abdominal general and gynecological surgery | I-III | Yes | PICO | -80 | 4 | HBDs | Yes | High |  |
| O’Neill 2020 | 40 | Pancreatectomy and hepatectomy | II | Yes | PICO | -80 * | 7 | GBDs | Yes | Some concerns |  |
| Pachowsky 2012 | 19 | Total hip arthroplasty | I | Yes | Prevena | -125 * | 5 | GBDs | Yes | Some concerns |  |
| Peterson 2021 | 110 | Caesarean section | II | Yes | PICO | -80 * | 7 | GBDs | Not reported | Low |  |
| Rashed 2021 | 104 | Sternotomy | I | Yes | VivanoTec | -125 | 5 | GBDs | Not reported | Some concerns |  |
| Ruhstaller 2017 | 119 | Caesarean section | II | Yes | Prevena | -125 * | 3 | GBDs | No | High |  |
| Sapci 2022 | 298 | Colorectal surgery | II-IV | Yes | Prevena | -125 | 6 | GBDs | No | Some concerns |  |
| Shen 2017 | 265 | Abdominal oncological resections | II | NR | NR | -125 | 4 | GBDs | No | High |  |
| Shields 2021 | 17 | Soft-tissue sarcoma surgery | I | NR | VAC | NR | NR | HBDs | No | Low |  |
| Shim 2018 | 51 | Hand surgery | I | NR | CuraVAC | -75 | 3 | GBDs | Yes | Some concerns |  |
| Stannard 2006 | 44 | Calcaneus, pilon, or tibial plateau fracture surgery | I-III | NR | VAC | -50 to -200 | NR | GBDs | Yes | Some concerns |  |
| Suh 2016 | 100 | SCIP flap harvest | I | Yes | CuraVac | -50 to -125 (cyclic) | 5 | GBDs | Not reported | Some concerns |  |
| Tuuli 2017 ¶ | 120 | Caesarean section | II | NR | PICO | -80* | 4 | ‘Standard’ | No (registration) | Some concerns |  |
| Tuuli 2020 | 1624 | Caesarean section | II | Yes | Prevena | -125 | 4 | GBDs | No | Some concerns |  |
| Vaddavalli 2022 | 50 | Lower extremity amputation because of peripheral arterial disease | I | NR | CCNPWT | NR | 6 | GBDs | No | Low |  |
| Wierdak 2021 | 75 | Open abdominal surgery | II-III | Yes | NANOVA | -125 * | 3 | NR | No | Some concerns |  |
| Wihbey 2018 | 166 | Caesarean section | II | Yes | Prevena | -125 * | 5 | GBDs | No | Some concerns |  |
| Witt-Majchrzak 2015 | 80 | CABG | I | Yes | PICO | -80 | 6 | GBDs | Yes | Some concerns |  |
| Yu 2017 | 72 | Saphenous vein harvest for CABG | I | Yes | Custom | -120 | 5 | GBDs | No | Some concerns |  |
| CABG, coronary artery bypass grafting; DIEAP, deep inferior artery perforator; GBDs, gauze-based dressings; HBDs, hydrocolloid-based dressings; NR, not reported; PAP, profunda artery perforator; SC, some concerns; SCIP, superficial circumflex artery perforator;  ¶ Conference abstract  * Pressure (mmHg) not mentioned in study  § Number of days or time of discharge, whichever came first | | | | | | | | | | | |

# **Appendix 5. Definitions of SSI**

| **Reference** | **SSI definitions** | **Other PWC definitions** |
| --- | --- | --- |
| **Andrianello 2021** | CDC classification | Seroma or hematoma: An incision opened attributable to  the presence of clinically relevant collections of blood or serous fluid, only in the absence of signs of infection. |
| **Arellano 2021** | CDC classification | NR |
| **Bertges 2021** | CDC classification | Wound dehiscence: skin separation requiring local wound care without meeting the criteria for an SSI.  Seroma or hematoma: collection of sterile fluid or blood under intact skin.  Wound necrosis: ischemia or necrosis of the skin edge that did not lead to an open wound but required local wound care. |
| **Borejsza-Wysocki 2021** | CDC classification | NR |
| **Bueno-Lledó 2021** | SSI: an infection that occurred at the site of a surgical incision or in an organ space within 30 days of the surgery. | Wound dehiscence: the splitting apart or rupturing of the margins of a previously closed wound along some or all of its length. |
| **Canton 2020** | NR | NR |
| **Chaboyer**  **2014** | CDC classification | NR |
| **Costa 2020** | CDC classification | NR |
| **Cooper 2022** | CDC classification | NR |
| **Crist 2017** | NR | NR |
| **Di Re 2021** | CDC classification | Superficial wound dehiscence: any separation of the skin not associated with an SSI. |
| **Engelhardt**  **2018** | Szilagyi classification  Grade I: only involvement of the skin (dermal infection);  Grade II: involvement of the subcutaneous tissue without reaching the vessels;  Grade III: involvement of the artery or bypass | NR |
| **Flynn 2020** | VICNISS classification  - Superficial: skin and subcutaneous tissue;  - Deep incisional: fascia and/or muscle  - Organ/space | NR |
| **Fogacci 2019** | NR | NR |
| **Gabriele 2021** | NR | NR |
| **Garg 2021** | NR | NR |
| **Gillespie 2015** | CDC classification | Any wound complication: patient who had dressings replaced before day 5 in the postoperative period. |
| **Gillespie 2021** | CDC classification | Bleeding: excessive wound bleeding postoperatively which can be identified by strikethrough on dressing.  Dehiscence: a surgical complication in which a wound splitting or rupture along surgical suture necessitating intervention.  Hematoma: collection of bloody fluid in the subcutaneous tissue due to failure of primary hemostasis or a bleeding diathesis.  Seroma: a serous fluid that accumulates in the subcutaneous tissue.  Hospital length of stay: the duration of a single episode of hospitalization calculated from admission day to the day patient was discharged.  Readmission: readmission after discharge within 30 days.  Pain: the presence of postoperative pain at or near the surgical wound area.  Reoperations: returned to surgery within 30 days of having CS procedure because of surgical wound complications.  Dressing related adverse events –blistering: when the epidermis is separated from the dermis and results from continued friction on the skin. This may be noted around dressing margins  Dressing related adverse events - itchiness or rash:  itchiness or rash caused by tape adhesive around the surgical wound area. |
| **Gök 2019** | NR | NR |
| **Gombert 2018** | Szilagyi classification  Grade I: limited to the dermis and included lymphatic leakage from the closed  Incision.  Grade II: infections extended to the subcutaneous skin layers.  Grade III: involvement of the arteries.  In cases of SSI deterioration, the patient was assigned a higher grade. | NR |
| **Gunatilake 2017** | NR | Surgical site occurrence: unanticipated local inflammatory response, prolonged drainage, fluid collection, dehiscence, and surgical site infection (SSI).  Surgical intervention: antimicrobials for SSI, surgical drainage of the incision, surgical incision packing, adjunctive negative-pressure therapy, debridement, or reoperation. |
| **Hasselman 2020** | CDC classification | Wound dehiscence was defined as the splitting apart or rupturing of the margins of a previously closed  wound along some or all of its length. |
| **Higuera-Rueda 2021** | CDC classification | Surgical site complication were defined as occurrence of superficial SSI, deep SSI, full-thickness skin dehiscence, seroma or hematoma requiring drainage, skin necrosis, or continuous drainage.  Skin dehiscence: a minimum 1mm wide separation at subcutaneous tissue level or deeper.  Seromas and hematomas: based on the nature of the fluid collection after drainage.  Continuous drainage: persistent wound drainage beyond the treatment period (>5-days), as identified at dressing removal. |
| **Hussamy 2019** | CDC classification | Wound complication: wound disruption or wound infection.  Wound disruption: the partial or complete opening of the deep subcutaneous space (dehiscence—underlying causes include seroma, hematoma), not to include only superficial skin separation.  Cellulitis required antibiotics and follow-up. |
| **Hyldig 2018** | Infection at incision site <30 days and treated with antibiotics | Deep surgical site infection: infection requiring surgery.  Minor dehiscence: a gap between the sides of the wound. |
| **Javed 2019** | CDC classification | NR |
| **Kacmaz 2022** | CDC classification and ASEPSIS Wound scoring system | NR |
| **Karlakki 2016** | NR | NR |
| **Keeney 2019** | NR | NR |
| **Lee 2017 CAR** | ASEPSIS score | NR |
| **Lee 2017 VASC** | CDC and Szilagyi classification | NR |
| **Leitao 2021** | CDC classification | NR |
| **Leon 2016** | NR | NR |
| **Li 2017** | CDC classification | Incision complications included wound infection, fat liquefaction, wound dehiscence, and wound effusion. |
| **Lopez 2023** | CDC classification | Surgical wound dehiscence was defined as the degree  of separation of the margins of the closed surgical incision, from superficial separation to the separation of the entire depth of the incision with exposure of body organs.  Seroma is a collection of serous fluid that forms in a cavity or space in the presence of a few red blood cells.  A surgical wound hematoma was defined as a  collection of blood that could be found in an organ, muscle, or under the skin after surgery. The |
| **Lychagin 2020** | Signs of gross wound infection were monitored by wound inspection and characteristic physical signs, i.e., increase in body temperature >37.5°C, wound discharge and characteristics of infection appearance. | NR |
| **Masden 2012** | Mild infection (erythema, inflammation) or severe infection (purulence, fever, leukocytosis) | Dehiscence (including sinus tracts, epidermolysis or frank dehiscence), or vascular compromise. |
| **Masters 2021** | CDC classification | NR |
| **Muller-Sloof 2018** | NR | NR |
| **Muller-Sloof 2022** | CDC classification | The secondary outcomes were formation of seroma and hematoma; the formation of these was ascertained by  needle aspiration. |
| **Murphy 2019** | CDC classification | NR |
| **Newman 2019** | Suture abscess defined as a localized area of redness induration and occasional purulence within the incision line that often required localized drainage [9], surgical site infection (SSI) defined as redness and edema around the surgical area that was significant enough to require intravenous antibiotics, | The primary outcome of the study was wound complications, which for the purposes of this study was defined as any one of the following [1]: drainage defined as fluid actively discharged from the incision which required a dressing change [2], cellulitis defined as any redness and edema around the surgical area that was significant enough to require oral antibiotics [3], blisters defined as the collection of fluid under the epidermis within 10 cm of the incision [4], hematoma defined as a collection of blood evidenced by ecchymosis surrounding the surgical area within 10 cm [5], skin necrosis defined as a darkened area of skin with no capillary refill within 10 cm of the incision [6], wound dehiscence defined as a wound in which the skin edges appeared together with good apposition that subsequently lost that apposition [7], non-healing wound defined as a wound in which the skin edges were never in apposition [8], suture abscess defined as a localized area of redness induration and occasional purulence within the incision line that often required localized drainage [9], surgical site infection (SSI) defined as redness and edema around the surgical area that was significant enough to require intravenous antibiotics, and [10] PJI defined using Musculoskeletal Infection Society criteria. |
| **O'Leary 2017** | CDC classification | NR |
| **O’Neill 2020** | CDC classification | NR |
| **Pachowsky**  **2012** | NR | This study only looks at post-surgical seroma. |
| **Peterson 2021** | CDC classification | NR |
| **Rashed 2021** | CDC classification  Deep sternal wound infection (DSWI) was defined if the infection involved at least the pectoral muscle fascia or deeper tissues in the incision, and the patient had at least one of the following: purulent drainage, organism isolated by culture –based microbiology testing, and signs of deep infection detected on gross anatomical or imaging test. | NR |
| **Ruhstaller 2017** | An SSI was defined as erythema and/or purulent drainage with or without fever that required antibiotic therapy. | An incision dehiscence or wound opening included any disruption of the skin closure with subcutaneous tissue exposure or a wound opening that required packing. |
| **Sapci 2022** | CDC classification | NR |
| **Shen 2017** | CDC classification | Wound dehiscence was defined as any spontaneous separation of the skin or fascia not associated with an SSI, seroma, or hematoma. Major morbidity was defined as any grade 3 or 4 serious adverse event as defined by the NIH’s Common Terminology Criteria for Adverse Events, v4.0. |
| **Shields 2021** | NR | NR |
| **Shim 2018** | NR | NR |
| **Stannard 2006** | Signs and symptoms consistent with infection | NR |
| **Suh 2016** | NR | NR |
| **Tuuli 2017** | NR | NR |
| **Tuuli 2020** | CDC classification | NR |
| **Vaddavalli 2022** | CDC classification | NR |
| **Wierdak 2021** | CDC classification | CWH: complete closure of the wound without any secretion from the wound |
| **Wihbey 2018** | CDC classification | Superficial, deep, or organ-space surgical site infection; wound dehiscence; seroma; or hematoma that occurred within 30 days of surgery |
| **Witt-Majchrzak 2015** | NR | A definition of the surgical site prepared by experts of the European Centre for Disease Prevention and Control (ECDC) and classification according  to El Oakley and Wright |
| **Yu 2017** | NR | NR |
| NR = not reported, PWC = postoperative wound complication | | |

# **Appendix 6. Forest plots secondary outcomes**

A. Wound dehiscence


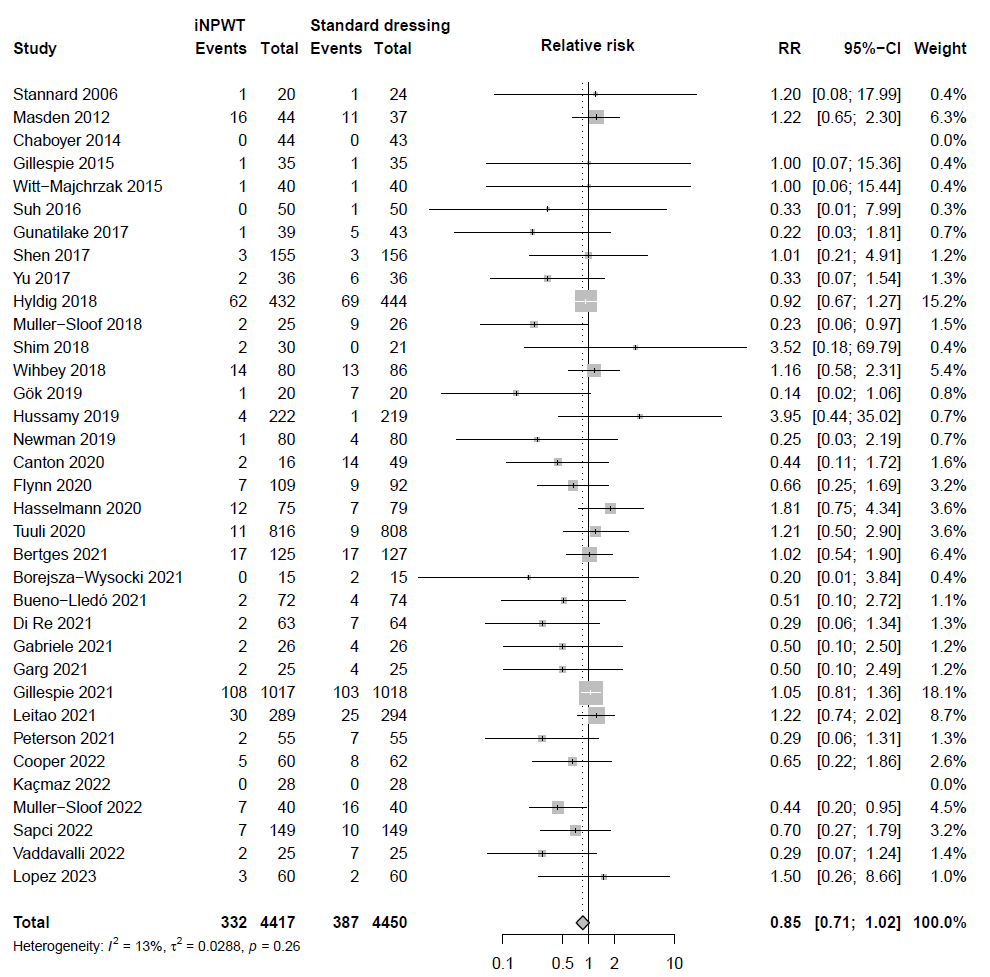


B. Reoperation


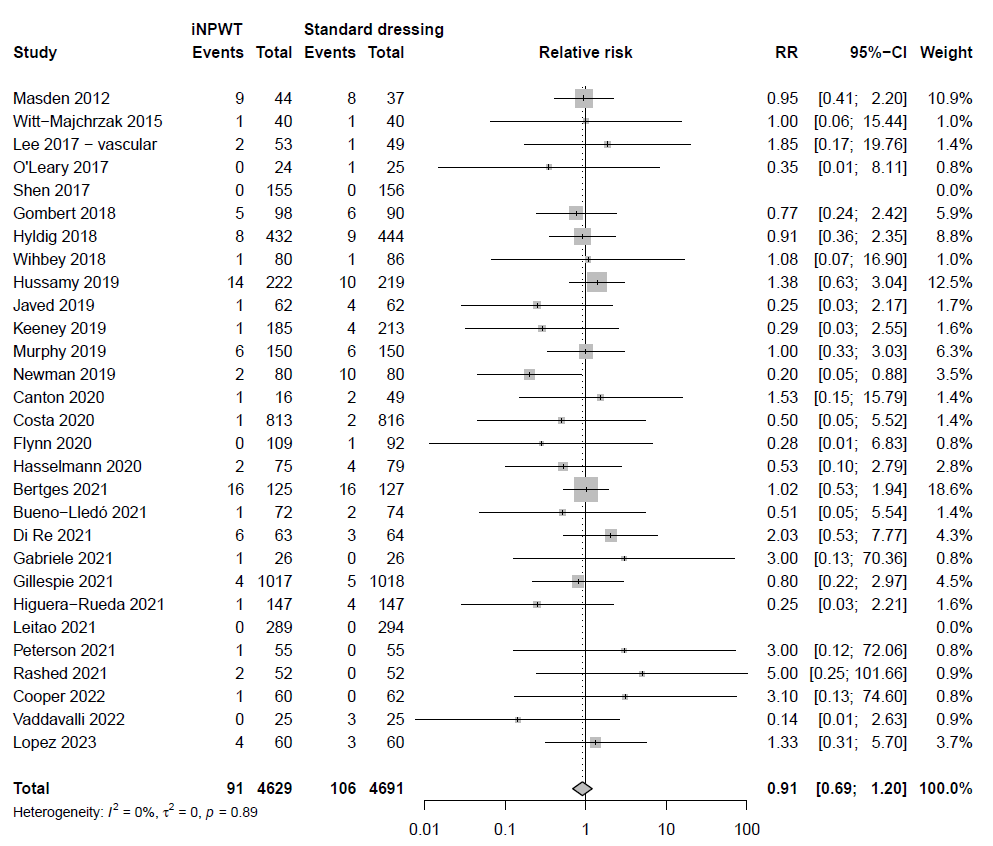


C. Seroma


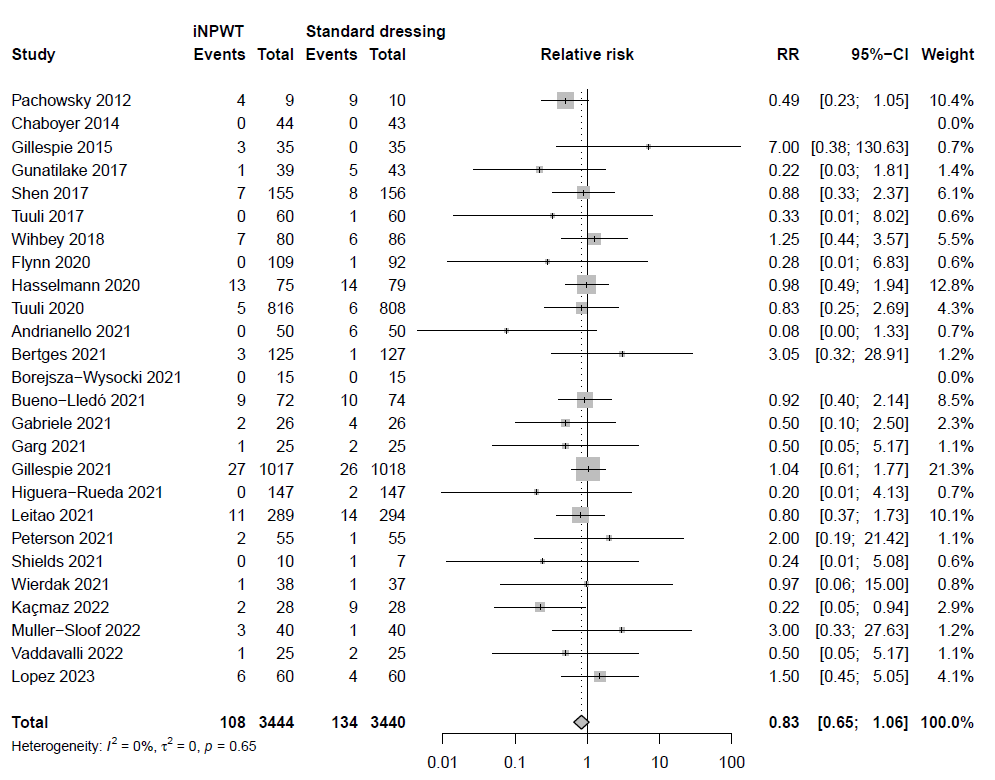


D. Hematoma


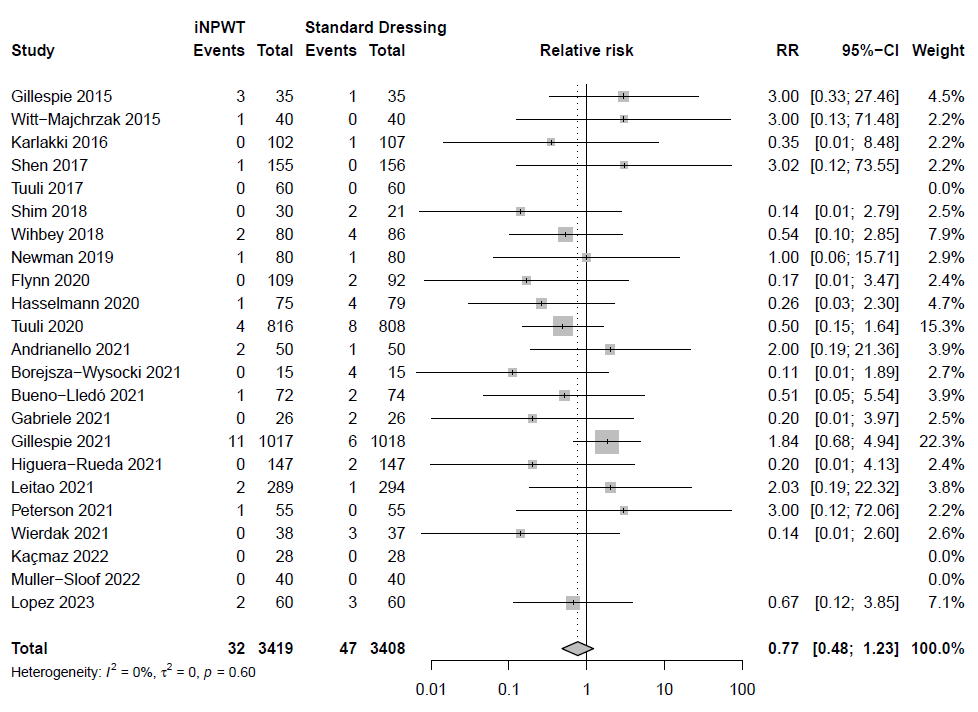


E. Mortality


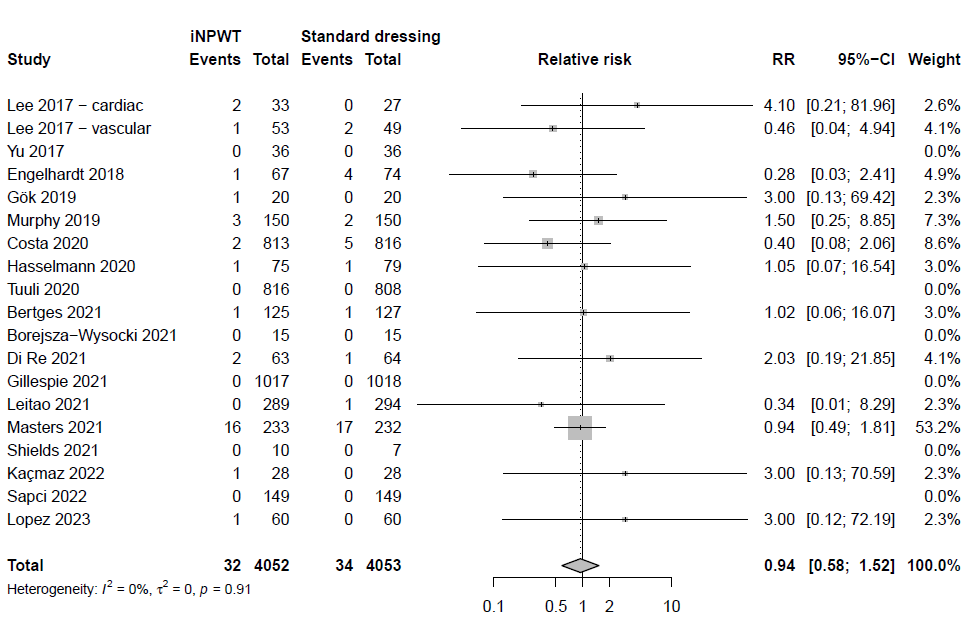


F. Readmission


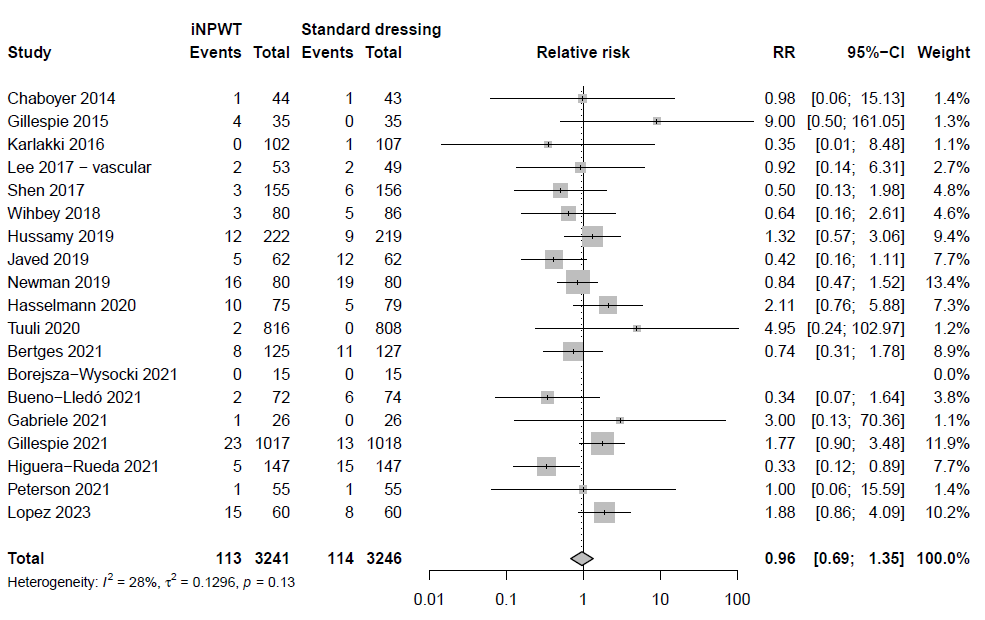


G. Skin blistering


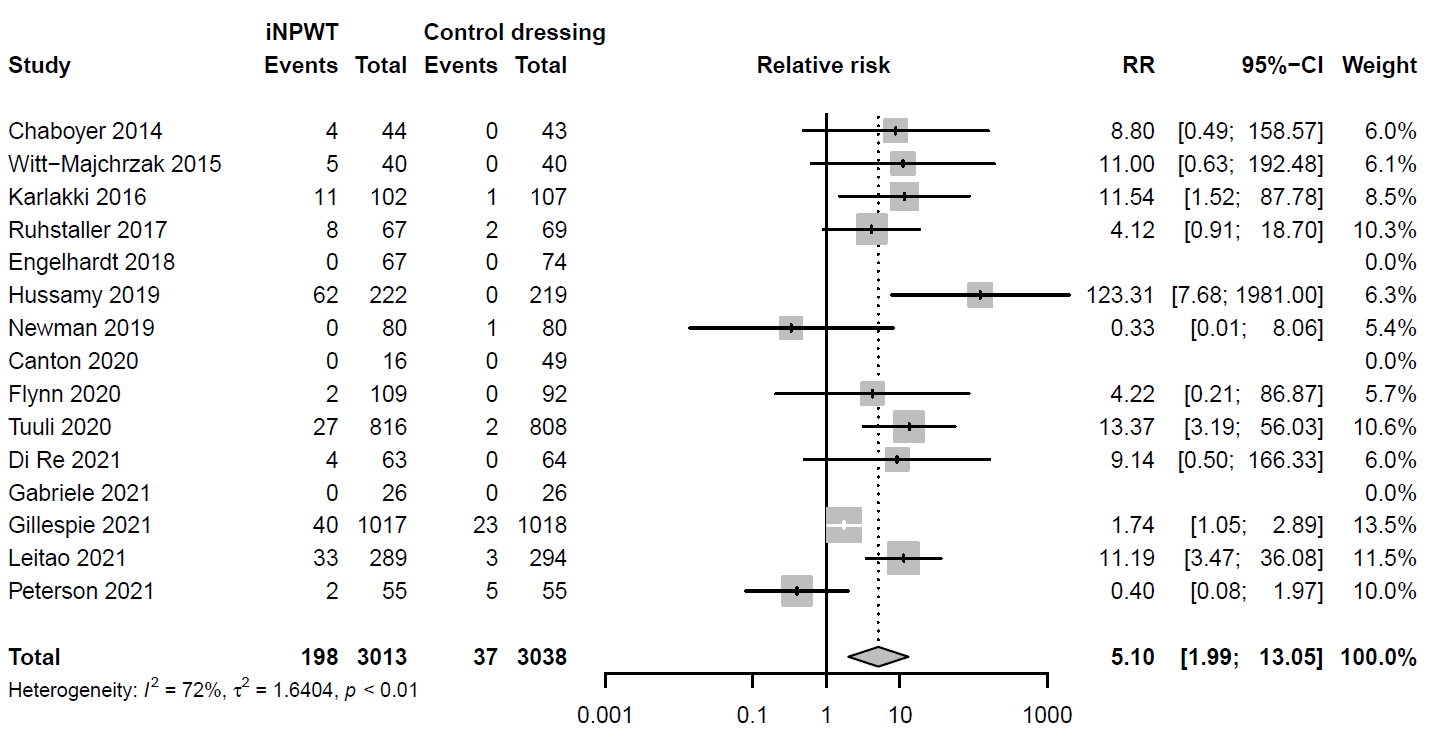


H. Necrosis


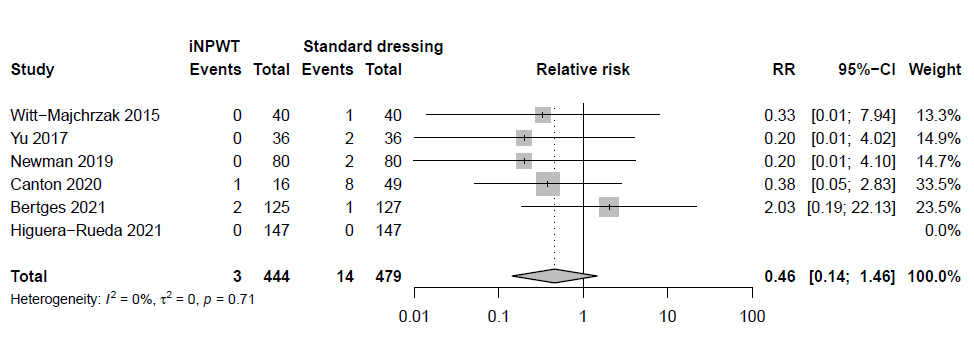


# **Appendix 7. Adverse events**

| **Study** |  | **Adverse events** |
| --- | --- | --- |
| Bertges 2021 | iNPWT | 15 adverse events (undefined, no significant adverse events were attributed to PREVENA) |
|  | Control | 19 adverse events |
| Borejsza-Wysocki 2021 | iNPWT | VAS-scores 1^st^ day: 4.0 ±1.1; 3^rd^ day: 2.5 ±.0.9; 5^th^ day: 2.3 ±1.1 |
|  | Control | VAS-scores 1^st^ day: 4.9 ±0.9; 3^rd^ day: 3.5 ±.0.9; 5^th^ day: 2.9 ±0.9 |
| Bueno-Lledó 2021 | iNPWT | No events attributable to iNPWT dressing (undefined) |
|  | Control | NR |
| Canton 2020 | iNPWT | No events of pain, discomfort, intolerance, rash, itching and blisters |
|  | Control | NR |
| Chaboyer 2014 | iNPWT | 4 skin blistering, 0 dermatitis, 1 bleeding |
|  | Control | 0 skin blistering, 1 dermatitis, 1 bleeding |
| Costa 2020 | iNPWT | 173 pain or tender |
|  | Control | 188 pain or tender |
| Di Re 2021 | iNPWT | 4 skin blistering |
|  | Control | 0 skin blistering |
| Engelhardt 2018 | iNPWT | No events (not defined) |
|  | Control | NR |
| Flynn 2020 | iNPWT | 2 skin blistering, 0 pain |
|  | Control | 0 skin blistering, 1 pain |
| Gabriele 2021 | iNPWT | 1 intolerance to treatment without complication (redding or blistering) |
|  | Control | NR |
| Garg 2021 | iNPWT | 0 pain |
|  | Control | NR |
| Gillespie 2015 | iNPWT | 8 bleeding |
|  | Control | 1 bleeding |
| Gillespie 2021 | iNPWT | 40 skin blistering, 10 dermatitis |
|  | Control | 23 skin blistering, 3 dermatitis |
| Gombert 2018 | iNPWT | No events of Szilagyi grade II-III SSIs, myocardial infarction, or hospital re-admission because of SSI) |
|  | Control | NR |
| Gunatilake 2017 | iNPWT | No serious events (not defined) |
|  | Control | No serious events (not defined) |
| Hasselmann 2020 | iNPWT | No events attributable to the iNPWT dressing (undefined) |
|  | Control | NR |
| Higuera-Rueda 2021 | iNPWT | 61 treatment-emergent adverse events (based on the Safety Analysis Set, type of event not further described) |
|  | Control | 71 treatment-emergent adverse events (based on the Safety Analysis Set, type of event not further described) |
| Hussamy 2019 | iNPWT | 62 skin blistering |
|  | Control | 0 skin blistering |
| Javed 2018 | iNPWT | No adverse skin reactions (not defined) |
|  | Control | No adverse skin reactions (not defined) |
| Karlakki 2016 | iNPWT | 11 skin blistering, 0 pain (no other adverse events described as outcome) |
|  | Control | 1 skin blistering, 1 pain |
| Lee 2017, cardiac | iNPWT | 1 rash or eczema, 2 itching, 2 pain (other adverse events outcomes were heaviness, weakness, stiffness, paraesthesia, numbness, burning, discolouration, oedema) |
|  | Control | 1 rash or eczema, 1 itching, 2 pain |
| Leitao 2021 | iNPWT | 33 skin blistering, 6 dermatitis, 6 pain (no other adverse events as outcome) |
|  | Control | 3 skin blistering, 4 dermatitis, 2 pain |
| Muller-Sloof 2018 | iNPWT | 0 dermatitis, 3 pain (no other adverse events as outcome) |
|  | Control | 0 dermatitis, 3 pain |
| Muller-Sloof 2022 | iNPWT | No serious adverse events occurred in relation to the study. |
|  | Control |  |
| Murphy 2019 | iNPWT | 6 bleeding. There were no reported adverse events from the NPWT dressing. |
|  | Control | 5 bleeding |
| Newman 2019 | iNPWT | 0 skin blistering (no other adverse events as outcome) |
|  | Control | 1 skin blistering |
| Peterson 2021 | iNPWT | 2 skin blistering |
|  | Control | 5 skin blistering |
| Ruhstaller 2017 | iNPWT | 8 skin blistering, 4 discomfort with device (1 removed), 5 malfunctions with device |
|  | Control | 2 skin blistering |
| Sapci 2022 | iNPWT | There were no adverse events associated with the iNPWT dressing. |
|  | Control | NR |
| Suh 2016 | iNPWT | No events of pain |
|  | Control | No events of pain |
| Tuuli 2017 | iNPWT | 2 skin reaction |
|  | Control | 0 skin reaction |
| Tuuli 2020 | iNPWT | 27 skin blistering, 10 dermatitis, 14 pain |
|  | Control | 2 skin blistering, 3 dermatitis, 1 pain |
| Witt-Majchrzak 2015 | iNPWT | 5 skin blistering |
|  | Control | 0 skin blistering |

# **Appendix 8. Forest plots sensitivity and subgroup analyses**

A. Type of Surgery – clustering orthopedic and trauma surgery


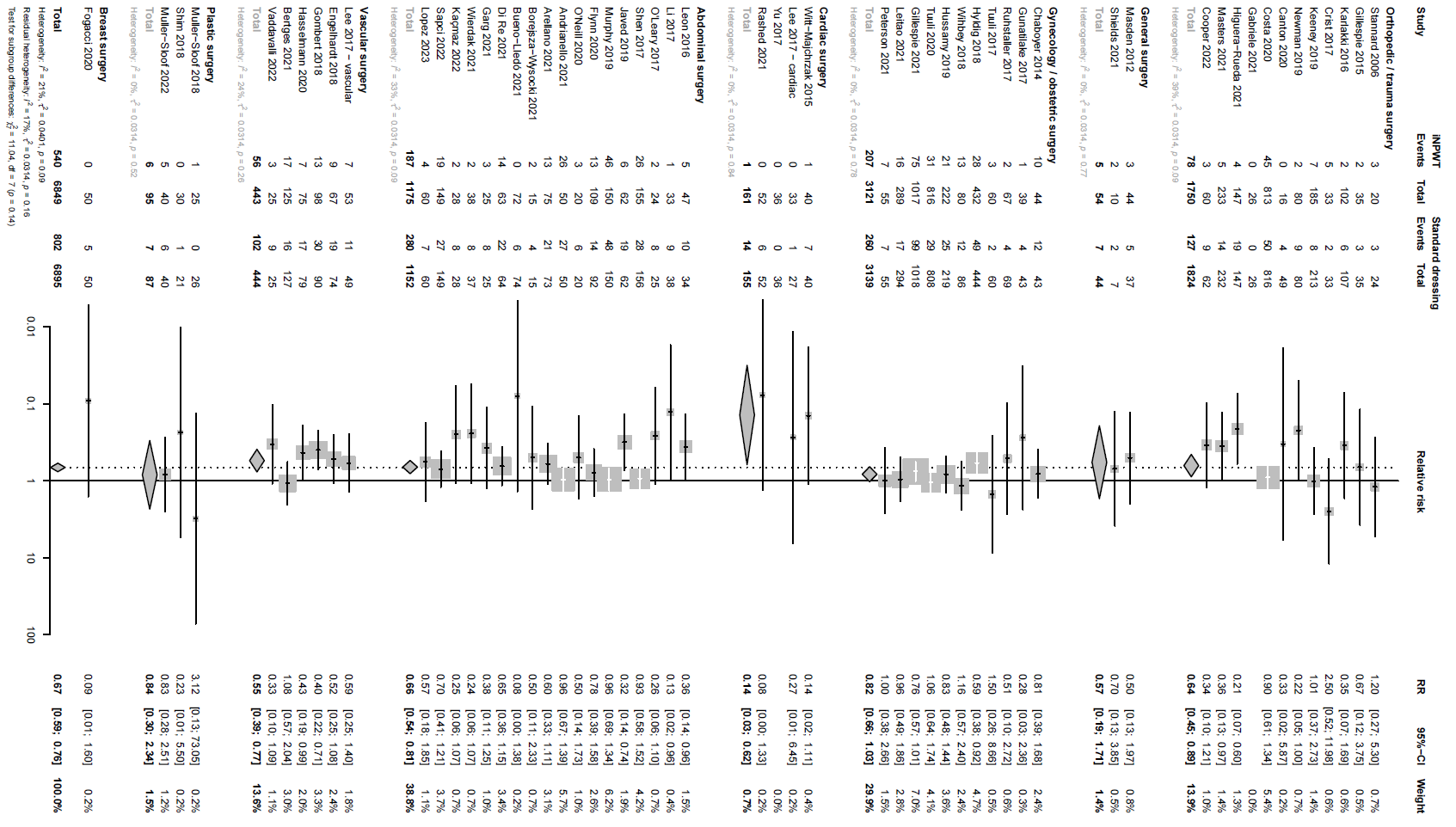


B. Type of Surgery – splitting orthopedic and trauma surgery


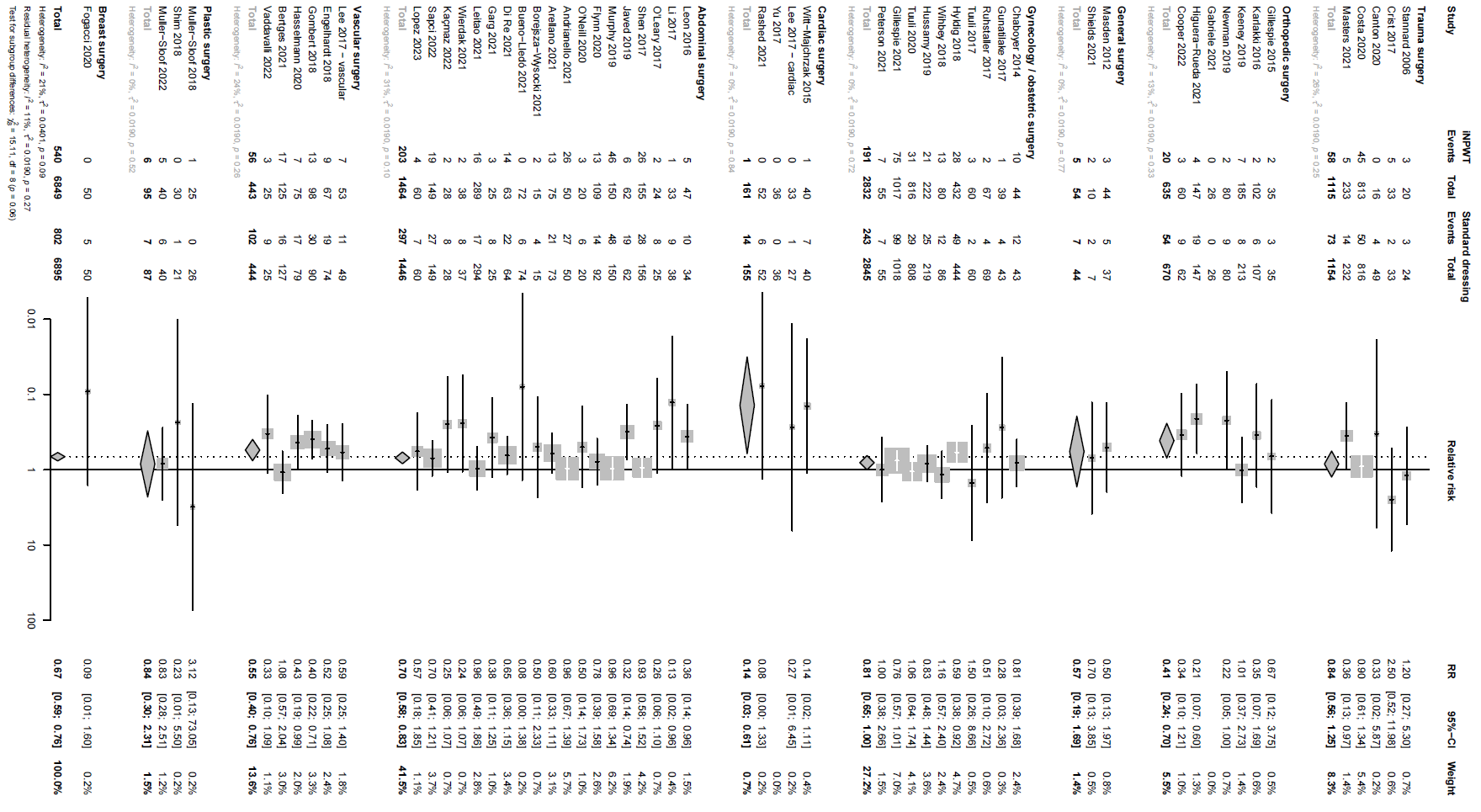


C. Industry involvement


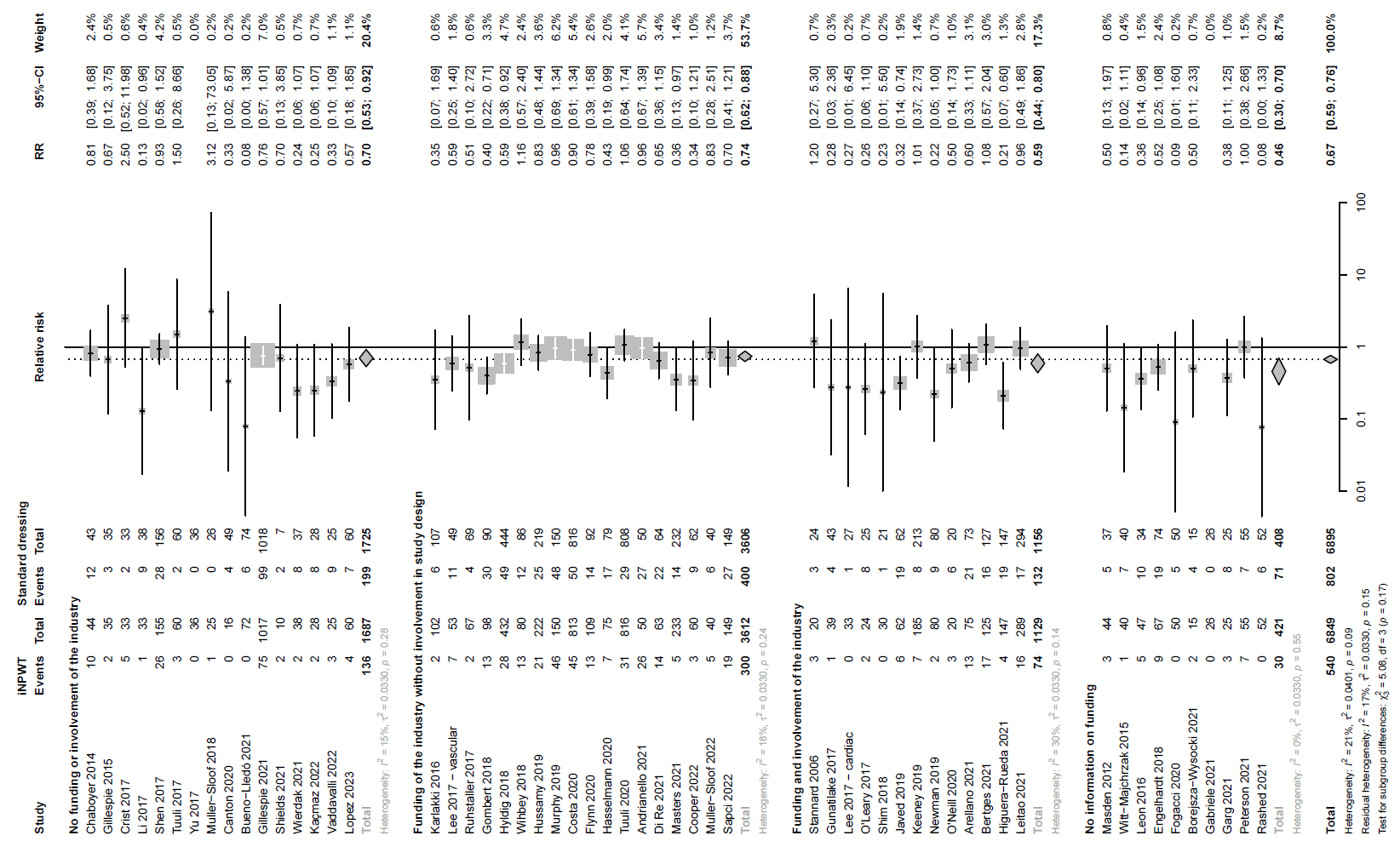


D. Pressure of device


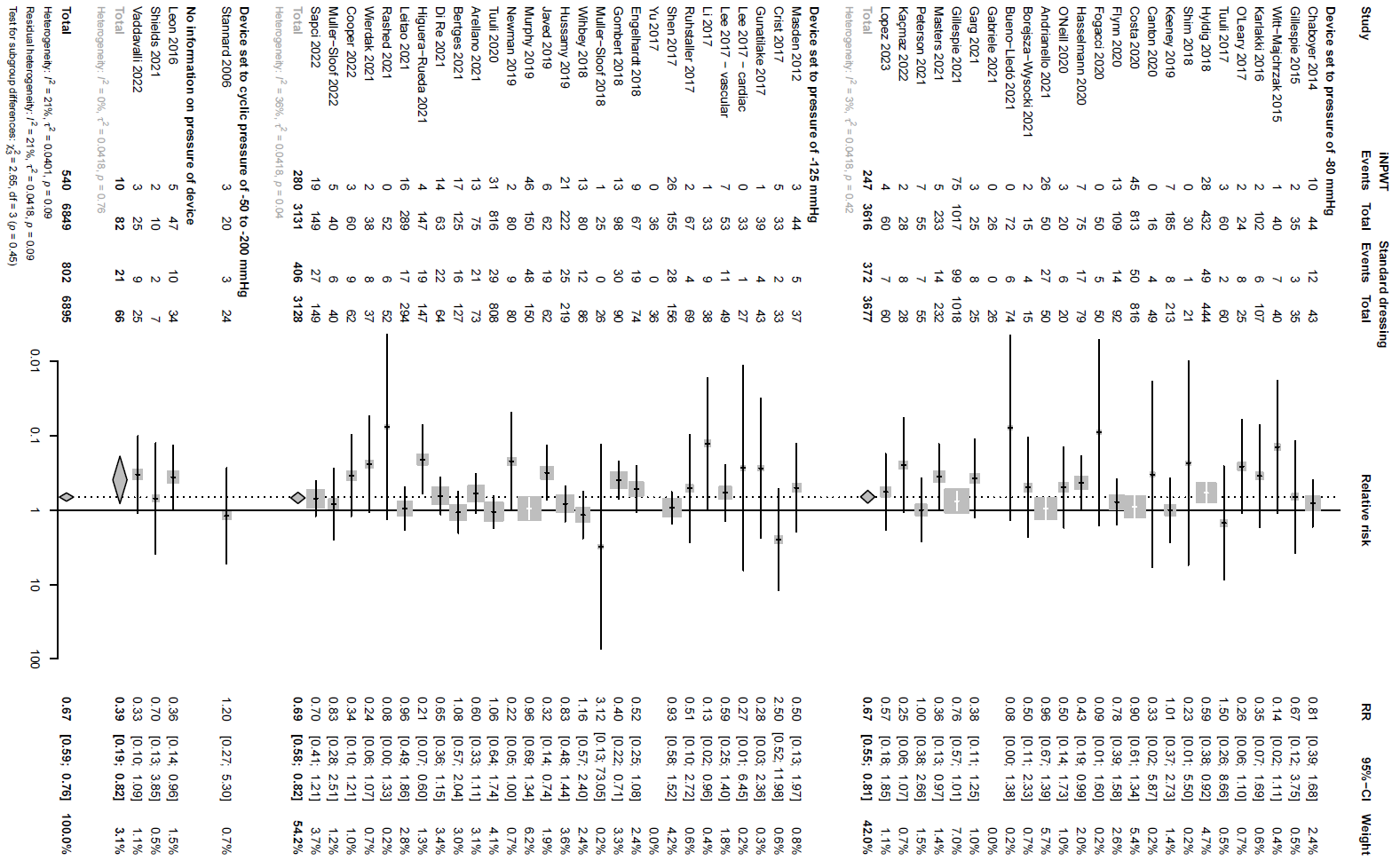


E. Risk of bias; low/some concerns vs. high risk of bias


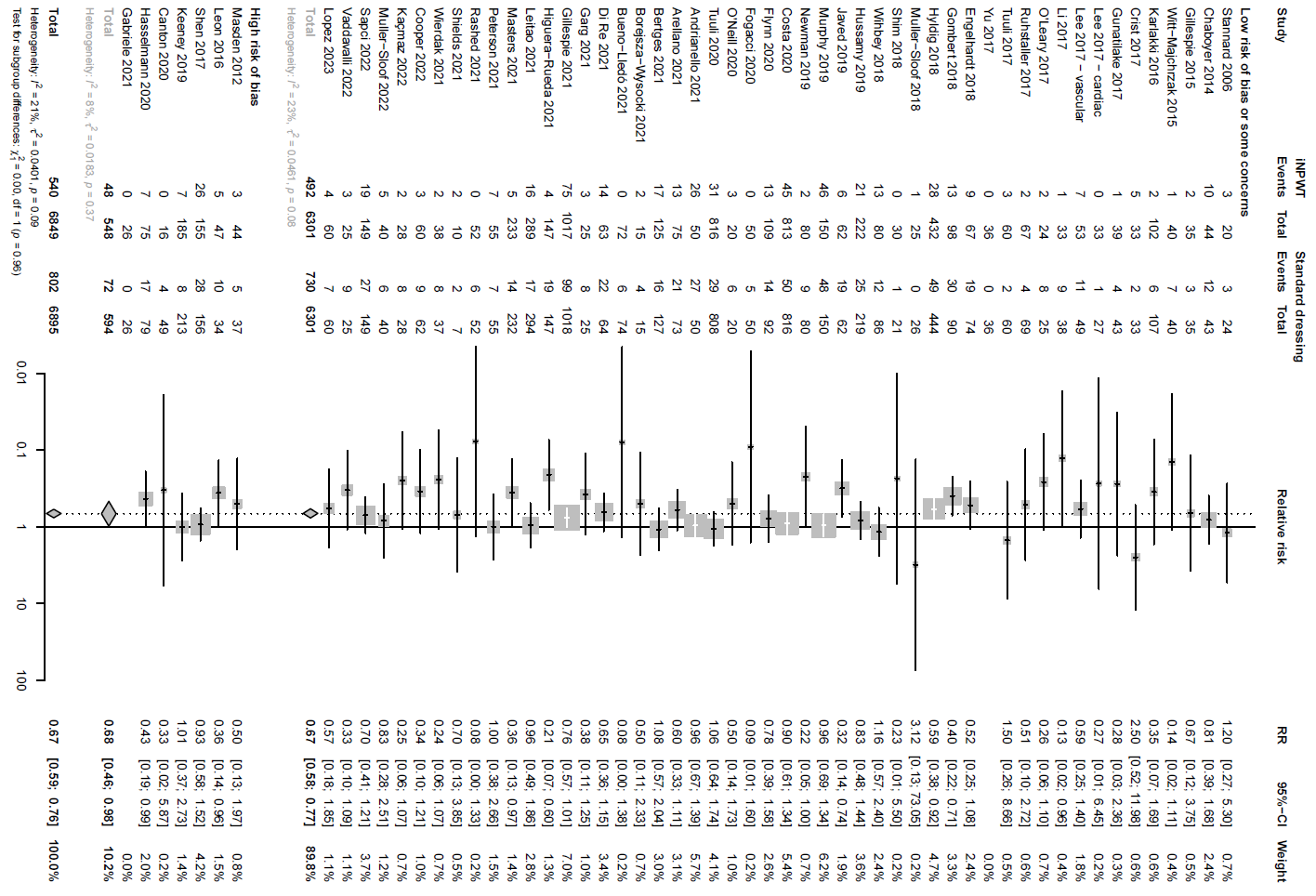


F. Risk of bias; low vs. some concerns vs. high risk of bias


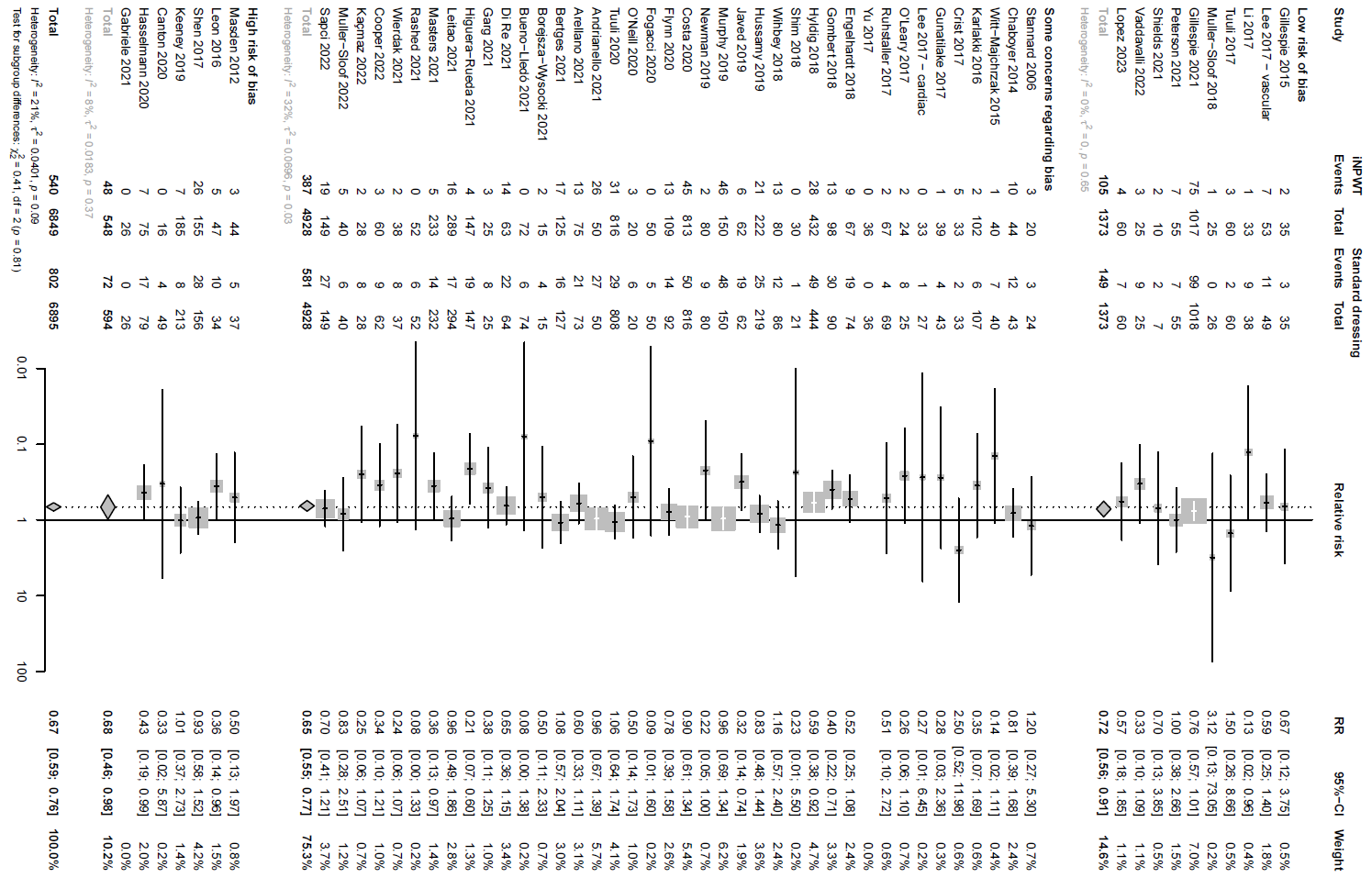


# **Appendix 9. Statements on industsry involvement**

| **Study** | **Statements on involvement of industry** | **Score** |
| --- | --- | --- |
| **Andrianello 2021** | Smith & Nephew Healthcare (Hull, UK) supplied the devices used for the study. The company was not involved in the analysis of the trial. | 2 |
| **Arellano 2021** | This study was sponsored by PREVENA Incision Management System, KCI, San Antonio, Texas, who provided the closed-incision negative-pressure therapy devices. | 3 |
| **Bertges 2021** | The present investigator-initiated study was funded by Acelity KCI (San Antonio, Texas). | 3 |
| **Borejsza-Wysocki 2021** | NR | 4 |
| **Bueno-Lledó 2021** | No commercial involvement | 1 |
| **Canton 2020** | No commercial involvement | 1 |
| **Chaboyer 2014** | No commercial involvement | 1 |
| **Cooper 2022** | This research was an investigator-initiated study that received limited support from Kinetic Concepts, Inc (San Antonio, TX; now 3M, Minneapolis, MN), who provided 62 surgical dressings for the study group at no cost to the participating institutions. No additional funding or support was received for study costs or research personnel. | 2 |
| **Costa 2020** | Smith and Nephew provided incisional negative pressure wound therapy dressings (PICO single use negative pressure wound therapy system) to recruiting centers. The sponsors had no role in the design and conduct of the study; collection, management, analysis, and interpretation of the data; preparation, review, or approval of the manuscript; and decision to submit the manuscript for publication. | 2 |
| **Crist 2017** | No commercial involvement | 1 |
| **Di Re 2021** | KCI (San Antonio, TX, USA) provided the NPWT dressings and devices for this study. No funding was received for this controlled trial. | 2 |
| **Engelhardt 2018** | NR | 4 |
| **Flynn 2020** | This trial was funded by Smith and Nephew, the company that produces the PICO dressing. Smith and Nephew received ongoing updates of all findings but did not have any active input or editorial power over the study protocol, day-to-day running of the trial or reporting of findings. | 2 |
| **Fogacci 2019** | NR | 4 |
| **Gabriele 2021** | NR | 4 |
| **Garg 2021** | NR | 4 |
| **Gillespie 2015** | No commercial involvement | 1 |
| **Gillespie 2021** | The trial was funded by a competitive peer reviewed grant (APP1081026) from the Australian National Health and Medical Research Council. The funders had no role in considering the study design or in the collection, analysis, or interpretation of data, the writing of the report, or the decision to submit the article for publication. | 1 |
| **Gök 2019** | NR | 4 |
| **Gombert 2018** | This investigator-initiated trial was funded by Acelity, San Antonio, TX, USA. The funder of this investigator-initiated trial had no role in study design, data collection, data analysis, data interpretation or writing of the article. | 2 |
| **Gunatilake 2017** | The study was sponsored by KCI, an Acelity Company, San Antonio, TX. | 3 |
| **Hasselmann 2020** | The research group received an unrestricted unconditional research grant of 15,550 USD and a donation of 100 PICO dressing kits from Smith and Nephew in 2013. | 2 |
| **Higuera-Rueda 2021** | The present investigation was funded by KCI-3M, San Antonio, TX. | 3 |
| **Hussamy 2019** | Study devices were provided by Kinetic Concepts Incorporated (San Antonio, Texas). This company had no input on the study design, collection, analysis, and interpretation of data, writing of the report, or the decision to submit the report for publication. | 2 |
| **Hyldig 2018** | Funding for this peer-reviewed, investigator-initiated clinical trial was provided by grants from the University of Southern Denmark, Odense University Hospital, the Region of Southern Denmark, Lundbeckfonden, and an unrestricted grant from the iNPWT device manufacturer Smith & Nephew (devices and operating funding). None of these sources of funds had an influence on study design, data collection, data analyses, interpretation of results or writing of the report. | 2 |
| **Javed 2019** | Funding: KCI/Acelity | 3 |
| **Kacmaz 2022** | The authors received specific funding for this work by the Erciyes University Scientific Research Projects Unit. | 1 |
| **Karlakki 2016** | The study was funded through a grant from Smith & Nephew UK, to cover the cost of NPWT dressings and data collection costs.  C. Whitall declares she has received payment from Smith & Nephew for other work  unrelated to this paper. | 2 |
| **Keeney 2019** | Our institution received research funding from Smith & Nephew Orthopaedics that was related to this study. | 3 |
| **Lee 2017, cardiac** | This work was supported by KCI USA Incorporated, an Acelity Company. | 3 |
| **Lee 2017, vascular** | Kinetic Concepts Inc (San Antonio, Tex) donated all NPWT devices but had no influence on study design, data collection, management or any input on publication. | 2 |
| **Leitao 2021** | The protocol was supported in part by KCI/Acelity. The role of the sponsor in the design, execution, analysis, reporting, and funding is fully disclosed. The sponsor reviewed the manuscript and provided general funding for research purposes. | 3 |
| **Leon 2016** | NR | 4 |
| **Li 2017** | No commercial involvement | 1 |
| **Lopez 2023** | This research did not receive any specific funding from any agencies in the public, commercial, or not-for-profit areas. | 1 |
| **Masden 2012** | Drs Attinger and Steinberg are consultants for Kinetic Concepts Incorporated | 4 |
| **Masters 2021** | J. Masters and J. Cook report institutional grants (paid to University of Oxford) from the Royal College of Surgeons of England/Dunhill Medical Trust research training fellowship, Smith & Nephew (device supply), and the NIHR Oxford Biomedical Research Centre (research infrastructure support), all related to this study. M. Costa reports institutional research grant funding (paid to University of Oxford) from the National Institute for Health Research (NIHR), the European Union (EU), the Royal College of Surgeons (RCS) England, and Smith & Nephew, not related to this study. | 2 |
| **Muller-Sloof 2018** | No commercial involvement | 1 |
| **Muller-Sloof 2022** | This study was funded in part by GD Medical (Houten, The Netherlands). Before commencing this study, both parties, the Department of Plastic Surgery at Radboud University Medical Center and GD Medical, stated in a written agreement (signed on March 31, 2017) that GD Medical would not have sight of the data or analysis of this study and would not have any influence on reporting and publication of the presented manuscript. | 2 |
| **Murphy 2019** | The study was funded by an industry grant from Kinetic Concepts Inc. (San Antonio, TX) in the amount of $87 000. The devices were also provided free of charge. KCI did not have any input on study design, data acquisition, date analysis, interpretation, and drafting of final manuscript. | 2 |
| **Newman 2019** | This study was funded by a research grant provided by KCI/Acelity Inc. (San Antonio, TX). | 3 |
| **O'Leary 2017** | Support was received from Smith and Nephew in the form of 25 PICO dressings. The authors were responsible for trial design, data analysis, and manuscript writing. The decision to publish the results of the trial was made together with the trial sponsor and study authors. | 3 |
| **O’Neill 2020** | The PICO incisional negative pressure wound therapy devices used in this study were provided Smith & Nephew, Hull, UK. | 3 |
| **Pachowsky 2012** | The PREVENA wound treatment system was provided by KCI free of charge. Matthias H. Brem gave scientific presentations for KCI. | 2 |
| **Peterson 2021** | NR | 4 |
| **Rashed 2021** | NR | 4 |
| **Ruhstaller 2017** | All study devices were provided by Acelity. The funding sources had no role in study design, data collection, or analysis. | 2 |
| **Sapci 2022** | Acelity/KCI provided the PrevenaTM Incision Management System used in this trial free of charge, without any other funding provided. Acelity/KCI had no input on data acquisition, analysis, or interpretation. | 2 |
| **Shen 2017** | No commercial involvement | 1 |
| **Shields 2021** | No benefits in any form have been received or will be received from a commercial party related directly or indirectly to the subject of this article. Dressings used in the study were funded through a charitable contribution from the local health board, NHS Greater Glasgow and Clyde Endowments (Registered Charity Number: SC005895). | 1 |
| **Shim 2018** | NR | 3 |
| **Stannard 2006** | It is hereby declared that first author, James P. Stannard, MD, is a Consultant for Kinetics Concepts, Inc. (KCI), the manufacturer of the Negative Pressure Wound Therapy device that is the subject of this manuscript. Additionally, KCI has provided financial assistance in the form of a grant for a clinical study relative to the VAC. | 3 |
| **Suh 2016** | NR | 4 |
| **Tuuli 2017** | NR in paper. On clinicaltrails.gov “Sponsor: Washington University School of Medicine” | 1 |
| **Tuuli 2020** | Acelity donated negative pressure devices and provided supplemental funding. The NIH and Acelity had no role in the design and conduct of the study; collection, management, analysis, and interpretation of the data; preparation, review, or approval of the manuscript; and decision to submit the manuscript for publication. Specifically, the funders had no right to veto publication or to control the decision regarding to which journal the manuscript was submitted. | 2 |
| **Vaddavalli 2022** | The authors received funding from Department of General Surgery, Post Graduate Institute of Medical Education and Research (PGIMER), Chandigarh, India. 160012. | 1 |
| **Wierdak 2021** | No commercial involvement | 1 |
| **Wihbey 2018** | The devices used in this study were provided by an unrestricted research grant from KCI Medical (San Antonio, Texas). KCI Medical had no role in the study design, data collection, or data analysis. Final results of the study were shared with the company before manuscript submission; however, the investigators were not bound to incorporate KCI’s comments. | 2 |
| **Witt-Majchrzak 2015** | NR | 4 |
| **Yu 2017** | No commercial involvement | 1 |
| 1: no industry funding or involvement  2: industry funding, without involvement in trial design  3: industry involvement in trial design  4: no information  NR: not reported | | |

# **Appendix 10. Bubble plot of intended duration of treatment**

Meta-regression showed that intended duration of treatment is not a significant effect size predictor (*p* = 0.69). Studies with longer intended duration of iNPWT treatment were not associated with a larger reduction in SSI, with a regression coefficient of 0.020. This means that for every additional intended day, the effect size (relative risk) is expected to rise by 0.020.


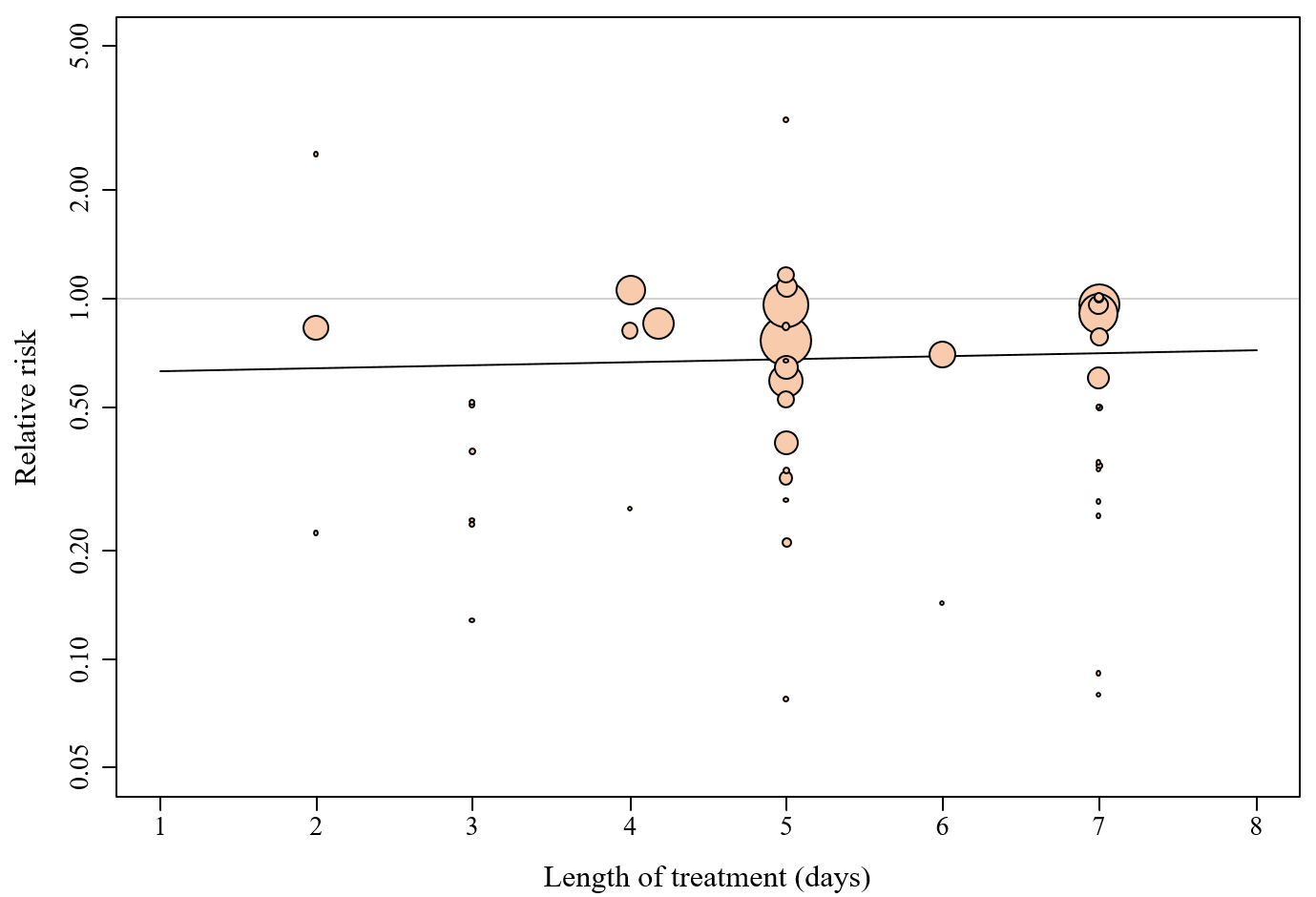


# **Appendix 11. Elaborate risk of bias assessment**


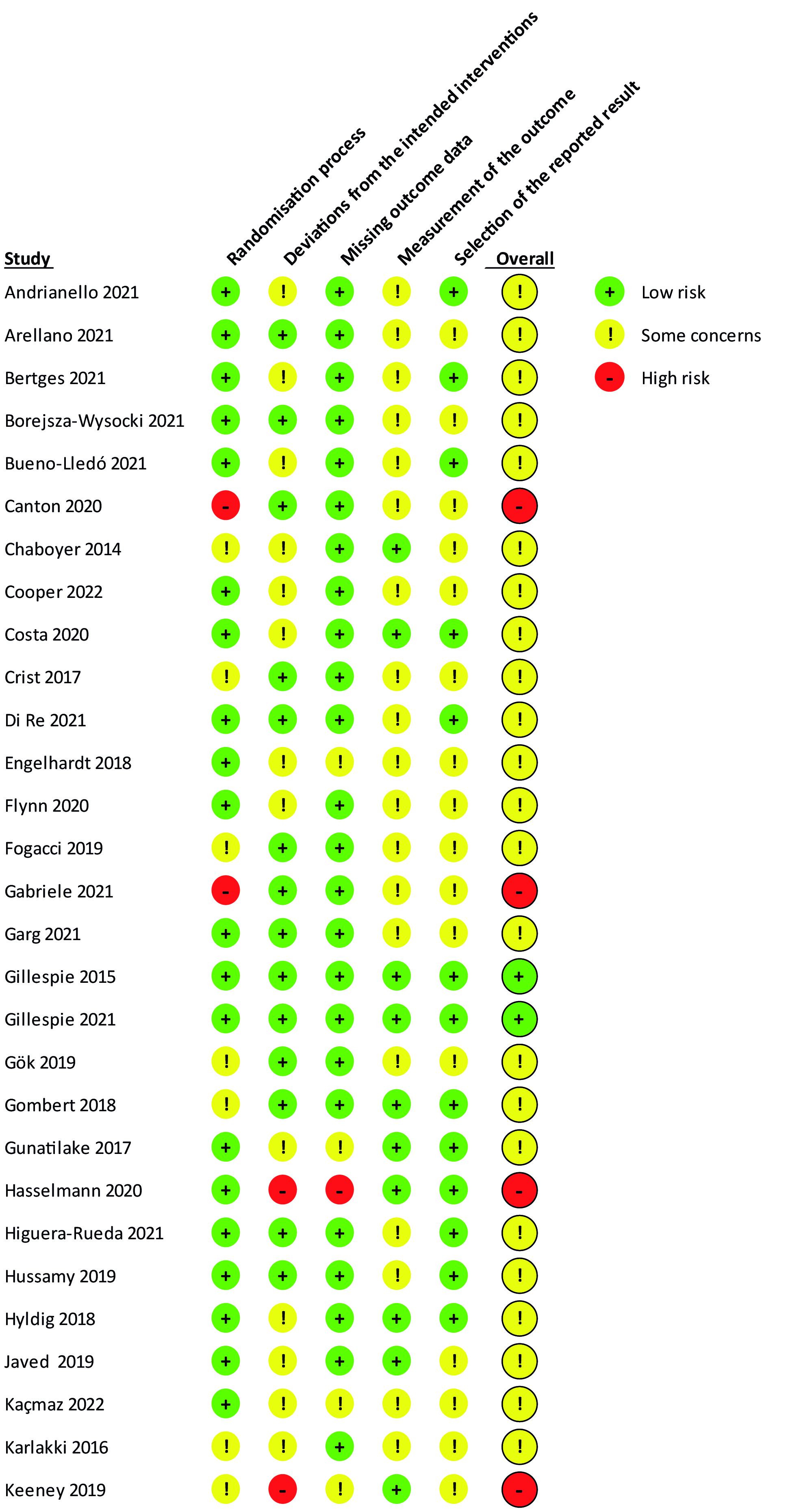

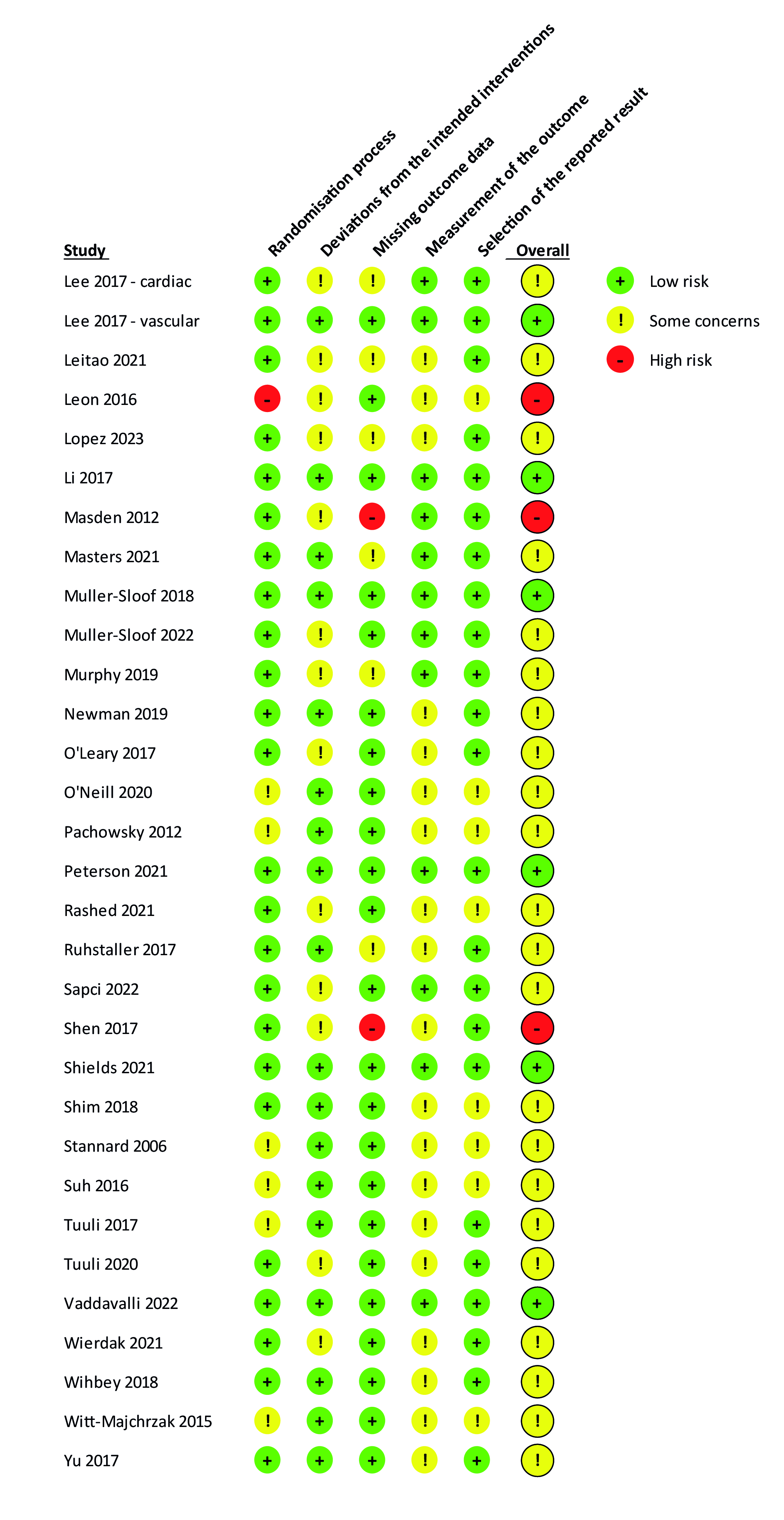


# **Appendix 12. Funnel plot for primary outcome**


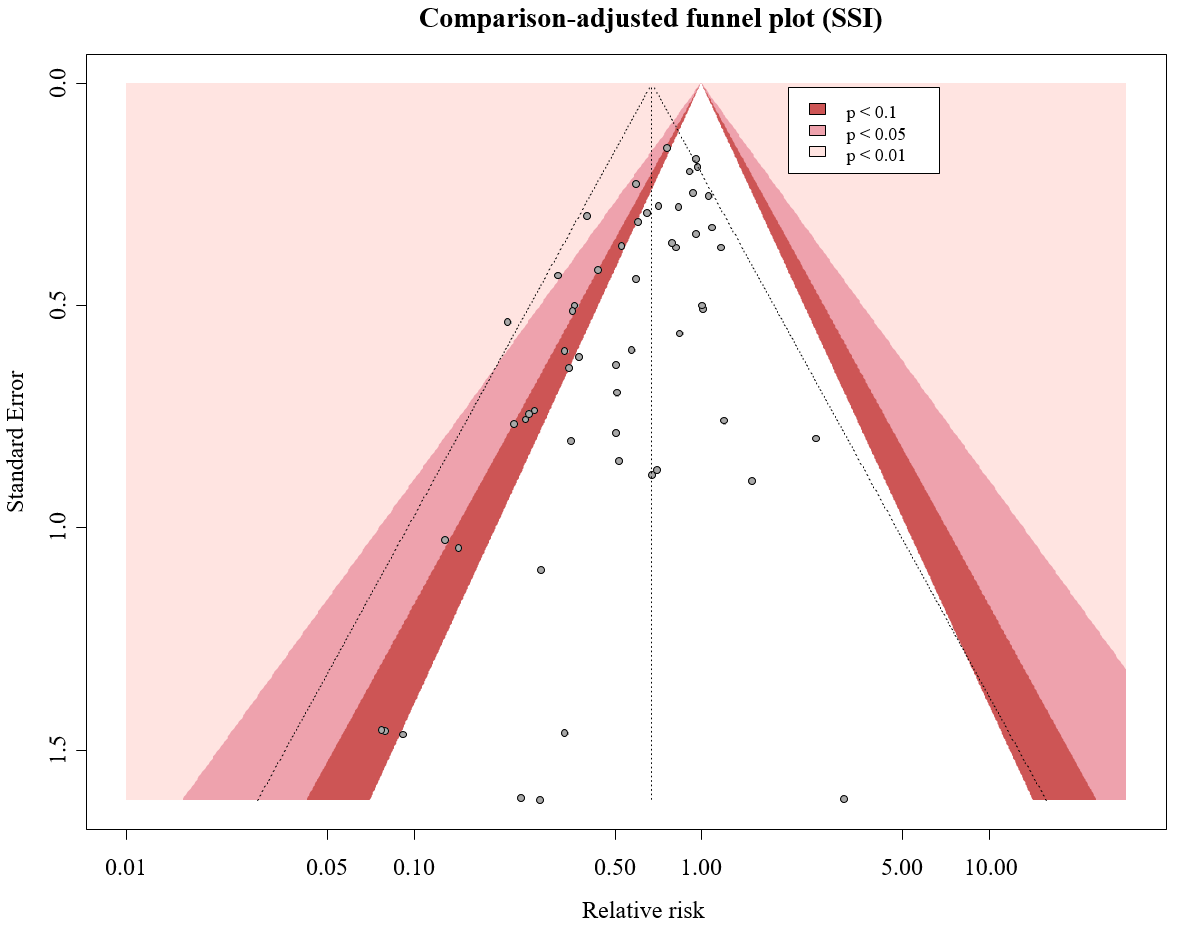

Supplement: Supplements_revision_2_clean_V5 [file mmc1.docx]
